# Cellular and molecular dysregulation of the esophageal epithelium in systemic sclerosis

**DOI:** 10.1172/jci.insight.195322

**Published:** 2026-07-16

**Authors:** Matthew Dapas, Margarette H. Clevenger, Hadijat-Kubura M. Makinde, Tyler Therron, Dustin A. Carlson, Mary Carns, Kathleen Aren, Cenfu Wei, Kainat Mian, Lutfiyya N. Muhammad, Carrie Richardson, Parambir S. Dulai, Monique Hinchcliff, John Pandolfino, Harris Perlman, Deborah R. Winter, Marie-Pier Tetreault

**Affiliations:** 1 Division of Rheumatology, Department of Medicine,; 2 Center for Human Immunobiology,; 3 Division of Gastroenterology & Hepatology, Department of Medicine,; 4 Kenneth C. Griffin Esophageal Center of Northwestern Medicine, and; 5 Department of Preventive Medicine, Northwestern University Feinberg School of Medicine, Chicago, Illinois, USA.; 6 Section of Rheumatology, Allergy & Immunology, Department of Medicine, Yale School of Medicine, New Haven, Connecticut, USA.

**Keywords:** Autoimmunity, Gastroenterology, Immunology, Cellular immune response, Rheumatology, Transcriptomics

## Abstract

Systemic sclerosis (SSc) is a rare autoimmune disease characterized by vasculopathy and fibrosis of the skin and internal organs. Individuals with SSc often suffer from chronic acid reflux and dysphagia due to loss of esophageal motility. To determine whether distinct changes in esophageal epithelial cells contribute to esophageal involvement in SSc, we investigated the stratified squamous esophageal epithelium from proximal and distal biopsies using single-cell RNA sequencing in individuals with SSc compared with those with gastroesophageal reflux disease (GERD) and healthy controls. Cellular and molecular changes in SSc were highly correlated with those seen in GERD, indicating they were secondary to reflux; however, their magnitudes were more pronounced in the proximal esophagus, suggesting that esophageal dysmotility leads to greater proximal acid exposure, which may contribute to aspiration. SSc-specific gene dysregulation implicated immunoregulatory pathways likely pertinent to pathogenic mechanisms. Ligand-receptor interaction analysis revealed enhanced profibrotic signaling between fibroblasts and epithelial cells in SSc. Cell type localization and SSc-specific changes were confirmed by spatial molecular imaging. By offering a comprehensive view of transcriptional dysregulation at single-cell resolution in human esophageal epithelial cells in SSc compared with GERD and healthy tissue, this work clarifies the state of epithelial cells in SSc-induced esophageal dysfunction.

## Abstract 2

Researchers shed new light on the changes that occur in esophageal epithelial cells in patients with systemic sclerosis.

## Introduction

Systemic sclerosis (SSc), also known as scleroderma, is an immune-mediated rheumatic disease of unknown etiology that causes fibrosis of the skin and internal organs. Over 90% of individuals with SSc report gastrointestinal dysfunction (1), with esophageal dysmotility being the most common gastrointestinal manifestation (2). Individuals with SSc and esophageal involvement typically suffer from chronic acid reflux and dysphagia due to loss of esophageal motility and are at much greater risk of esophageal stricture and Barrett’s esophagus (1). Esophageal dysmotility is also associated with more severe skin and lung disease in SSc (3). The prevalence of esophageal involvement was nearly twice as high in individuals with SSc who died within 5 years of disease diagnosis (4).

There are multiple, interrelated mechanisms that may cause esophageal dysmotility in SSc. Vascular damage and/or neurogenic impairment can lead to smooth muscle atrophy (5), which is the predominant esophageal pathology in autopsies of patients with SSc (6). Inflammatory signatures (7), absence of anti-centromere antibodies (8), and presence of anti–topoisomerase I antibodies are associated with esophageal pathologies as well (1). Research efforts are complicated by the significant clinical and molecular heterogeneity observed between SSc patients (9) and the difficulty in distinguishing between effects of autoimmune processes and secondary factors such as severe gastroesophageal reflux disease (GERD). Consequently, the pathogenesis of esophageal disease in SSc remains poorly understood.

Functional investigations of SSc esophageal involvement have primarily focused on peristalsis or contractions of the muscularis propria (10). However, the mucosa is no less essential for normal esophageal transport (11, 12), and recent studies have suggested that esophageal epithelial cells (EECs) may play a more central role in the pathogenesis of SSc esophageal involvement than previously thought (13). Mouse models with epithelial cell–specific knockout of *Fli1*, an Ets transcription factor implicated in SSc pathogenesis (14), spontaneously develop dermal and esophageal fibrosis and interstitial lung disease (15). Importantly, the resultant lung disease is mediated by T cell autoimmunity, but fibrosis of the skin and esophagus persists in mice additionally lacking mature T and B cells, suggesting that the fibrosis is principally driven by the epithelia (13, 15).

Most studies implicating epithelial cells in SSc esophageal involvement have been performed in mice or in vitro, and molecular analyses in humans have so far been limited to bulk tissues (7). Furthermore, single-cell gene expression studies performed in other diseases of the esophagus, including squamous cell carcinoma (16, 17) and allergic eosinophilic esophagitis (18, 19), have identified substantial cellular and molecular changes in EECs compared with healthy tissue, such as significant expansion of non-proliferative suprabasal cells (18, 19). Therefore, to comprehensively investigate the effects of SSc on the esophageal epithelium, we performed single-cell RNA sequencing (scRNA-seq) of esophageal mucosal biopsies from SSc patients with clinically significant esophageal involvement and compared EEC distributions and gene expression signatures with those in non-SSc individuals with GERD and healthy controls (HCs). We further validated cell type localization and SSc effects using spatial molecular imaging from independent SSc and HC samples. By examining gene expression profiles from human tissue at single-cell resolution, this work sheds essential light on the cellular roots of esophageal dysfunction in SSc by clarifying the pathogenic role of the squamous epithelium, one of the most integral tissues supporting healthy esophageal function.

## Results

Whole-tissue scRNA-seq was performed on paired proximal and distal mucosal biopsies from 10 patients with SSc, 4 non-SSc patients with GERD, and 6 HCs. All participants but one in each group were female (Table 1). SSc patients were significantly older than HCs (54.9 vs. 27.0 years; *P* < 0.001). Among the SSc patients, 6 had the limited cutaneous subtype, 3 had the diffuse cutaneous subtype, and 1 had sine scleroderma with no cutaneous symptoms (Supplemental Table 1; supplemental material available online with this article; https://doi.org/10.1172/jci.insight.195322DS1). Four SSc patients had absent esophageal contractility, 4 had ineffective motility, 1 had esophagogastric junction outflow obstruction, and 1 had type II achalasia, according to the Chicago Classification schema for esophageal motility disorders (20). Median SSc disease duration at the time of biopsy was 72 months (interquartile range = 63–149). All SSc patients had positive serum antinuclear antibodies and impaired esophageal motility. Following sample-level quality control (Supplemental Table 2), 39 of 40 samples were retained for analysis, including 19 from the proximal esophagus and 20 from the distal esophagus. Four additional sets of proximal and distal biopsies were collected for spatial molecular imaging from 2 SSc patients with diminished esophageal contractility and 2 HCs (Supplemental Table 3).

### Characterization of esophageal mucosal cell populations.

**Figure 1:**
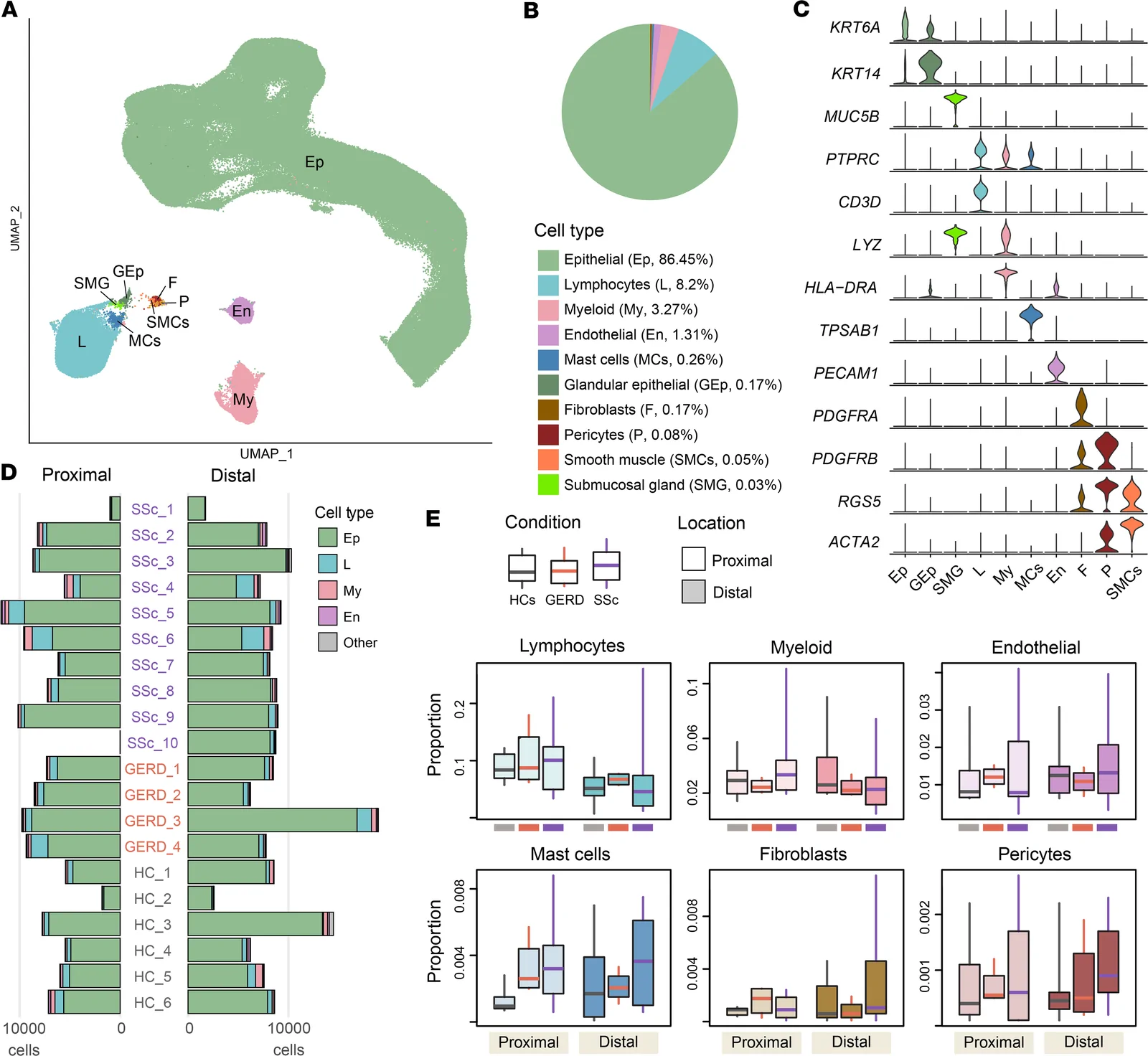
scRNA-seq sample composition by cell type. (**A**) Integrated UMAP embedding of all cells (*n* = 306,372) labeled by cell type. (**B**) Proportion of cells by cell type. (**C**) Expression by cell type of canonical markers. (**D**) Sample-wise (*n* = 39) distribution of major cell types. (**E**) Proportion of major non-epithelial cell types by condition and biopsy location. Box whiskers extend to range of values.

A total of 306,372 esophageal cells (7,856 mean cells per sample) were retained for cell clustering and gene expression analysis. Each cell type was identified according to the expression of established cell-specific gene markers including *KRT6A*, *KRT13*, and *KRT15* for epithelial cells (Figure 1, A–D, Supplemental Table 4, and Supplemental Figure 1). Epithelial cells constituted the bulk of the sample (*n* = 264,858; 86.4%), followed by lymphoid cells (*n* = 25,129; 8.2%), myeloid cells (*n* = 10,019; 3.3%), endothelial cells (*n* = 4,027; 1.3%), and all other cell types (*n* = 2,339; 0.8%). We observed heterogeneity in non-epithelial cell type proportions by condition and biopsy location (Figure 1E).

In a previous gene expression study of the SSc esophagus, Taroni and colleagues identified clusters of SSc patients with distinct gene expression signatures using microarrays (7). To determine whether these “intrinsic” inflammatory, proliferative, and non-inflammatory subsets could be delineated among our SSc patients, we analyzed their reported expression signatures in our single-cell data. We found that patient-level expression of these gene sets correlated strongly with cell type proportions (Supplemental Figure 2, A and B). In particular, scores for the inflammatory subset signature were most strongly associated with lymphoid and myeloid cell proportions (upregulated genes: *r*_s_ = 0.78 and *r*_s_ = 0.60, respectively; downregulated genes: *r*_s_ = –0.75 and *r*_s_ = –0.77, respectively; Supplemental Figure 2B). Based on cell type proportions alone, samples from the same SSc patients tended to cluster together (Supplemental Figure 2C), with cell type composition significantly more correlated between biopsy locations within individuals (mean *r* = 0.89) than between all other sample pairs (mean *r* = 0.77, *P* = 0.004; Supplemental Figure 2D). Hierarchical clustering of patients based on pseudobulk expression of intrinsic subset genes (7) revealed inter-patient heterogeneity that was likewise associated with cell type proportions, with proximal and distal samples from the same patients clustering together (Supplemental Figure 2E). While inter-patient heterogeneity was evident, not all patients clearly segregated into the discrete subsets defined by Taroni et al. (7).

### Significant loss of superficial EECs in SSc.

**Figure 2:**
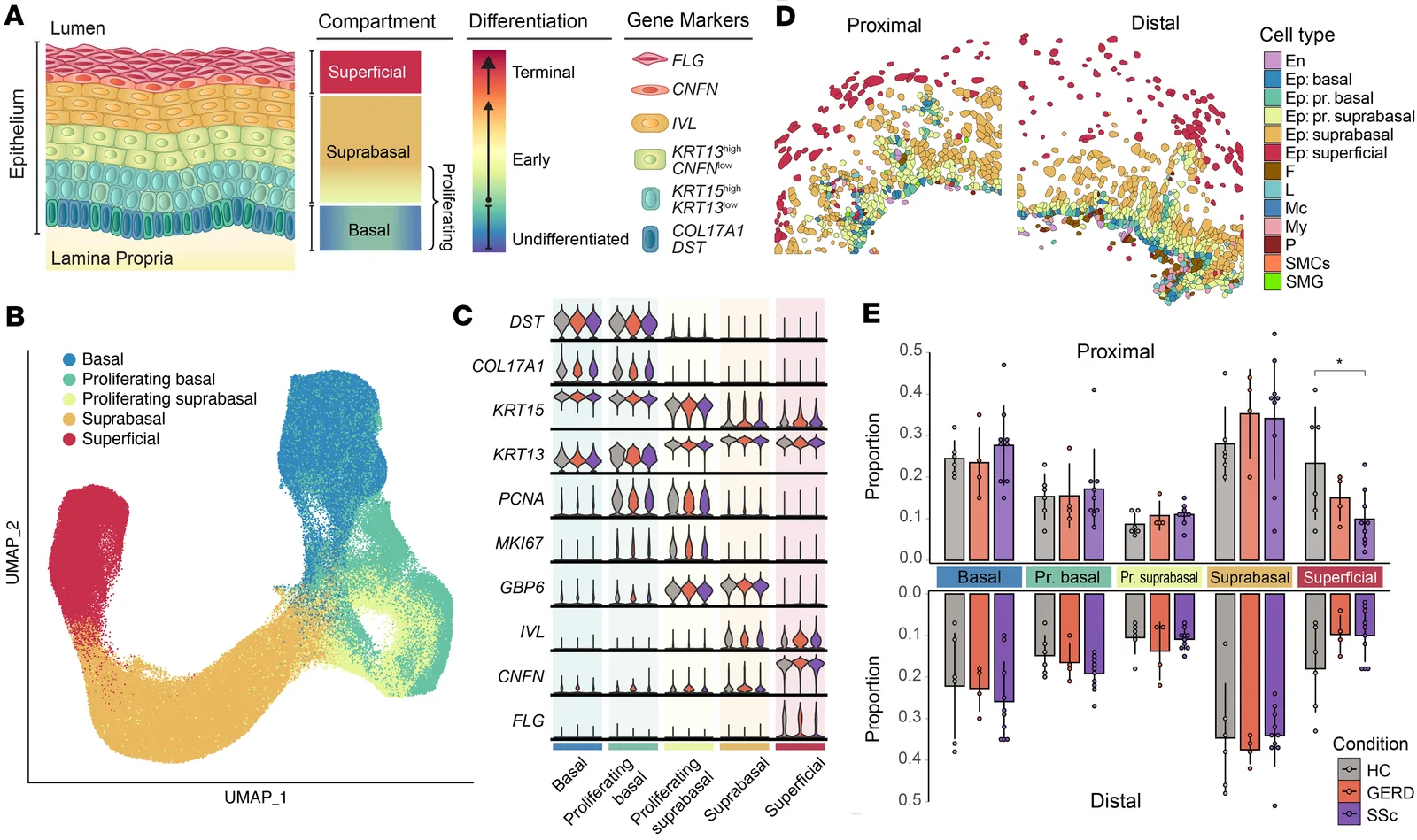
Landscape of esophageal epithelial cells. (**A**) Histological summary of the human esophageal epithelium, adapted from Clevenger et al. (19). Alternatively shaded cells within the same layer represent proliferating cells. Note that the layers of replicating cells not attached to the basement membrane, labeled here as proliferating suprabasal cells, are sometimes referred to as epibasal cells (19). (**B**) Integrated UMAP embedding of all esophageal epithelial cells (EECs; *n* = 230,720) labeled by epithelial compartment. (**C**) Expression of EEC compartment and cell cycle markers by condition. (**D**) CosMx spatial molecular imaging of proximal and distal esophageal mucosal tissue in SSc with absent contractility, annotated using label transfer from the scRNA-seq data. (**E**) Proportions of cells by condition, EEC compartment, and biopsy location. The bars denote the mean values, the vertical lines the standard deviations, and the points the individual sample proportions. Pairwise differences across conditions were evaluated statistically using mixed-effects modeling of associations of single cells (MASC) (96). *P < 0.05.

Following isolation of the epithelial cell cluster and additional quality control (Supplemental Figure 3, A–E), we characterized the remaining 230,720 EECs according to their differentiation and cell cycling states (Figure 2A). We classified the EECs into 5 primary compartments: basal (*n* = 55,818; 24.2%), proliferating basal (*n* = 36,439; 15.8%), proliferating suprabasal (*n* = 25,314; 11.0%), suprabasal (*n* = 82,283; 35.6%), and superficial (*n* = 30,866; 13.4%), based on relative expression of canonical epithelial genes and cell cycle markers (Figure 2, B and C, and Supplemental Figure 3, F–K). Spatial localization of the annotated single-cell clusters was confirmed using spatial molecular imaging (Figure 2D and Supplemental Figure 4). Roughly 40% of basal cells and 24% of suprabasal cells were proliferating, and the fractions of cells that were proliferating did not differ by disease (Figure 2E). Proportions of basal and suprabasal cells were not significantly different across conditions, but the proportion of proximal superficial cells in SSc (sample mean = 9.9%) was substantially lower than that in HCs (sample mean = 23.3%, *P* = 0.017) (Figure 2E).

### EEC gene dysregulation was predominantly in superficial cells and highly correlated between SSc and GERD.

**Figure 3:**
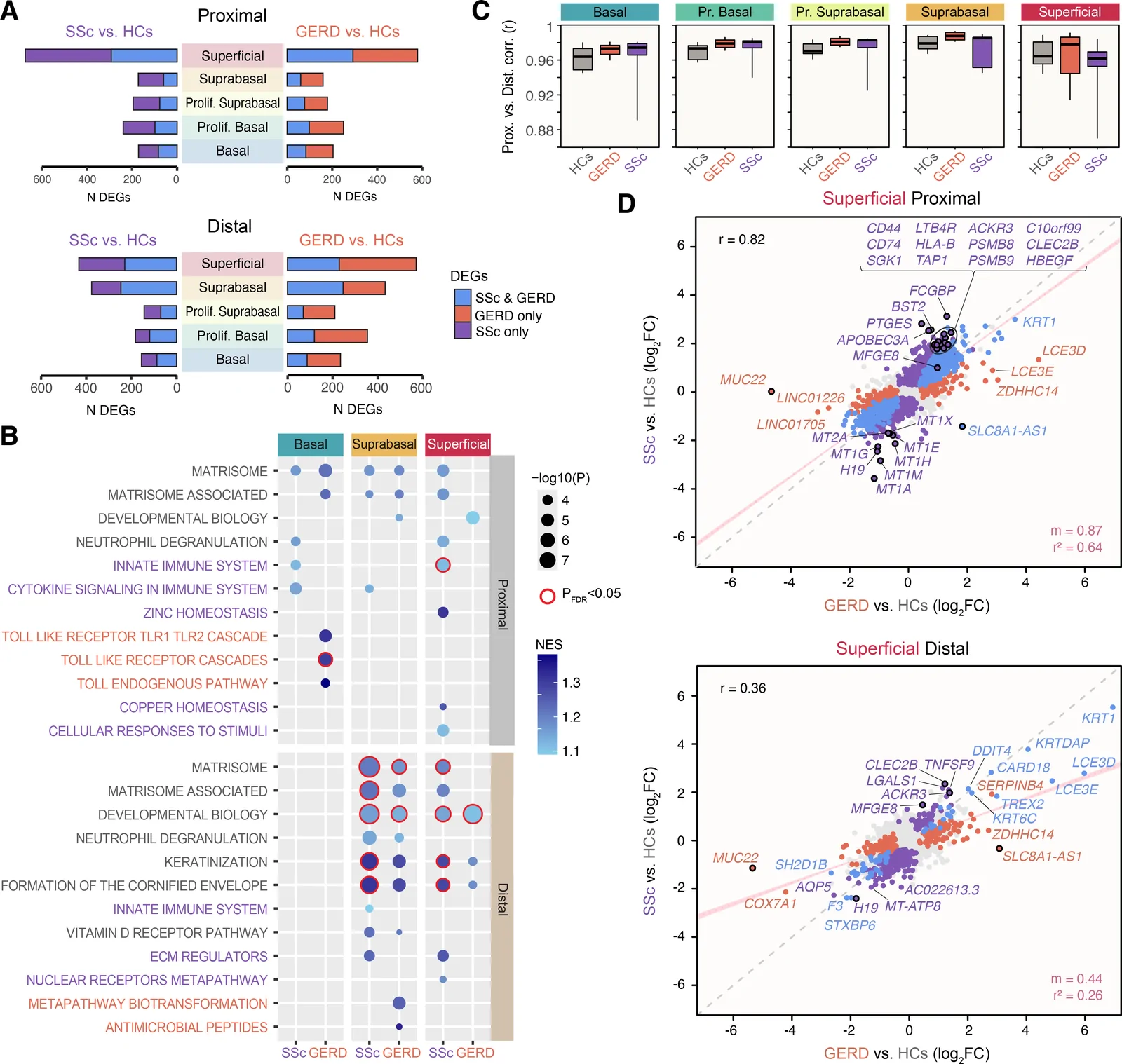
Differential gene expression between conditions in EECs. (**A**) The number of significantly differentially expressed genes at the single-cell level (Wilcoxon’s rank-sum) with |log_2_ fold change| > 0.1 is shown for SSc versus HCs (purple) and GERD versus HCs (orange), by epithelial compartment and biopsy location. Blue bars account for genes that were differentially expressed in both SSc and GERD compared with HCs. (**B**) Gene set enrichment analysis results, showing all pathways enriched with *P* < 0.001 (unadjusted; 100,000 permutations) for SSc versus HCs (purple) and GERD versus HCs (orange), split by non-proliferating epithelial compartment and biopsy location. Points circled in red indicate statistical significance after adjustment for the number of tested pathways (*P*_FDR_ < 0.05). (**C**) The distribution of intra-sample EEC gene expression correlations for the 2,000 most variable genes between proximal and distal biopsies is shown by epithelial compartment and condition. Box whiskers extend to range of values. (**D**) Pseudobulk gene expression differences in log_2_ fold change (log_2_FC) are shown for SSc versus HCs (*y* axis) against GERD versus HCs (*x* axis) within the superficial compartment in the proximal and distal esophagus regions. Significant DEGs in both conditions are highlighted in blue, significant DEGs in SSC only are highlighted in purple, and significant DEGs in GERD only are highlighted in orange (edgeR) (97). The correlation between comparisons is shown at upper left, and the slope and coefficient of determination for the modeled linear regression with intercept = 0 are displayed at lower right. Trendlines with 95% confidence intervals are plotted in pink, and dashed gray lines denote where *y* = *x*. Encircled points highlight disease-specific DEGs that are referenced in the main text and plotted in Supplemental Figure 5H, which shows the distribution of expression aggregated by sample in the superficial compartment across conditions and biopsy locations.

Most differentially expressed genes (DEGs) were only differentially expressed in one epithelial compartment (Supplemental Figure 5A), and differential gene expression between conditions was most pronounced in superficial EECs (Figure 3A). At the single-cell level, 674 and 434 genes were significantly differentially expressed between SSc and HCs in the superficial compartment in the proximal and distal regions, respectively, compared with 171 proximal and 157 distal non-proliferating basal DEGs and 172 proximal and 377 distal non-proliferating suprabasal DEGs (Supplemental Tables 5 and 6). To more robustly characterize gene expression differences between conditions (21), we performed pseudobulk differential expression analysis by aggregating single-cell RNA counts per sample within EEC compartments. Overall, pseudobulk gene expression patterns clustered by compartment rather than by condition or biopsy location, and most inter-sample expression heterogeneity was observed in superficial cells (Supplemental Figure 5B). Significant differences in gene expression between conditions were largely limited to superficial cells, suggesting that the apical cell layers of the esophageal epithelium are most affected by SSc (Supplemental Figure 5, C and D). There were 3,572 genes differentially expressed between SSc and HCs with FDR *q* < 0.05 in the superficial compartment, compared with just 232 total in all other compartments (Supplemental Table 7).

The differential expression observed in SSc was strongly correlated with the changes seen in GERD, with many of the same genes significantly dysregulated in both SSc and GERD compared with HCs. Overall correlations in pseudobulk log_2_ fold change between SSc versus HCs and GERD versus HCs were consistently strong, ranging between *r* = 0.51 and *r* = 0.62 across EEC compartments (Supplemental Table 7). Depending on the compartment and biopsy location, 22%–44% of single-cell DEGs in SSc or GERD were differentially expressed in both conditions (Figure 3A and Supplemental Table 5). Consequently, the single-cell DEGs in SSc and GERD were significantly enriched for some of the same pathways, particularly those related to the extracellular matrix and keratinization (Figure 3B). These included matrisome (suprabasal, distal; superficial, distal) and matrisome-associated genes (suprabasal, distal); cornified envelope formation (suprabasal, distal; superficial, distal) and keratinization genes (suprabasal, distal; superficial, distal); and developmental biology–related genes (suprabasal, distal; superficial, distal). The leading edges of these significant pathways, which comprised the genes driving their enrichment, included shared protein families, including serine protease inhibitors (serpins), keratins, small proline-rich proteins (SPRRs), and S100 proteins (Supplemental Table 8). Among the most enriched pathways specific to SSc were those related to the regulation of metal and immune homeostasis. In particular, innate immune system genes were uniquely enriched in SSc compared with HCs in superficial EECs of the proximal esophagus.

Overall gene expression levels were highly similar between esophageal biopsy locations, with mean intra-individual correlations grouped by condition and epithelial compartment ranging from *r* = 0.95 to *r* = 0.99 for the 2,000 most variable genes (Figure 3C and Supplemental Table 9). However, we did observe some notable differences in differential gene expression patterns by biopsy location. For example, single-cell differential expression in the suprabasal compartment was significantly greater in the distal esophagus than in the proximal esophagus for both SSc (172 proximal vs. 377 distal, *P* = 2.5 × 10^–20^) and GERD (159 proximal vs. 434 distal, *P* = 1.1 × 10^–32^) (Figure 3A and Supplemental Figure 5A). Furthermore, at the single-cell level, there were more unique DEGs in SSc versus HCs in the proximal esophagus (897 proximal vs. 806 distal, *P* = 7.0 × 10^–2^), but the opposite was true in GERD versus HCs (910 proximal vs. 1,075 distal, *P* = 1.6 × 10^–5^) (Figure 3A). Similarly, within the superficial compartment, where most differential expression was observed, GERD exhibited greater relative changes in pseudobulk gene expression in the distal esophagus than in the proximal region, as indicated by the decreased slope between fold changes (*m* = 0.44 vs. 0.87; Figure 3D and Supplemental Table 10). The superficial compartment also featured the greatest range and lowest overall values of intra-individual gene expression correlations between biopsy locations, particularly in SSc (Figure 3C). These differences by biopsy location suggest that the relative disease effect on the proximal esophagus compared with the distal esophagus is greater within SSc than within GERD.

More genes were significantly differentially expressed in SSc than in GERD in the pseudobulk analysis, but in terms of relative fold change versus HCs, we observed greater expression changes in GERD (Figure 3D). To determine whether the greater number of SSc samples was driving this discrepancy, we performed all possible sample permutations with equal numbers of SSc and GERD samples (*n* = 4) and repeated differential gene expression testing to more directly compare the relative number of DEGs in both conditions. In superficial cells from the proximal esophagus, there were more DEGs in SSc in 60% of permutations, but the relative fold-change differences were greater in GERD in 61% of permutations (Supplemental Figure 5, E and F, and Supplemental Table 11). In the distal region, there were more DEGs in GERD in 62% of permutations, and the relative fold-change differences were greater in GERD in all permutations. The correlations between SSc and GERD in terms of relative gene expression changes were consistently much higher in the proximal region permutations than in the distal region (Supplemental Figure 5G). The permutation results confirmed that the differences in gene expression observed in SSc relative to GERD were not simply due to differences in sample size. In summary, the superficial epithelium is most altered in SSc, exhibiting transcriptional changes similar to those in GERD, but with relatively greater effect in the proximal esophagus compared with distal.

### SSc-specific gene dysregulation points to immunoregulatory pathways.

Among the most upregulated SSc-specific genes (Figure 3D and Supplemental Figure 5H) were genes related to inflammation (*PTGES*, *MFGE8*) (22, 23), innate immune response (*FCGBP*, *BST2*, *CD44*, *APOBEC3A*) (24–27), immune cell migration (*C10orf99*, *LTB4R*, *ACKR3*) (28–30), antigen presentation (*HLA-B*, *CD74*, *TAP1*, *PSMB8*, *PSMB9*) (31), natural killer cell activation (*CLEC2B*) (32), and fibroproliferation (*SGK1*, *HBEGF*) (33, 34). Four of the top 5 and 8 of the top 20 most downregulated SSc-specific genes by fold change in superficial EECs in the proximal esophagus were metallothioneins (*MT1A*, *MT1E*, *MT1F*, *MT1G*, *MT1H*, *MT1M*, *MT1X*, *MT2A*). The metallothioneins constituted the bulk of 3 pathways uniquely enriched in SSc with *P* less than 0.001: zinc homeostasis, copper homeostasis, and cellular responses to stimuli (Figure 3B). The most downregulated SSc-specific gene across both the proximal and distal esophagus was the *H19* long non-coding RNA. Interestingly, the most downregulated gene by fold change in GERD, *MUC22*, was not differentially expressed in SSc. One gene, *SLC8A1-AS1*, was significantly differentially expressed in both GERD and SSc, but in opposite directions (Figure 3D, Supplemental Figure 5H, and Supplemental Table 10).

### Transcription factors enriched in proximal SSc esophagus.

We next examined whether DEGs in SSc superficial cells were significantly enriched for targets of specific transcription factors. Among genes differentially expressed in SSc compared with GERD, the target genes of IRF1 were significantly enriched (Supplemental Table 12). IRF1 targets were significantly enriched among all proximal superficial DEGs (adjusted *P* value [*P*_adj_] = 0.021) and among only those that were downregulated (*P*_adj_ = 0.0024). However, *IRF1* was not itself differentially expressed in superficial cells in SSc compared with GERD. Compared with HCs, 3 transcription factors were significantly enriched in SSc in the proximal esophagus: MYC (*P*_adj_ = 2.1 × 10^–7^) and E2F4 (*P*_adj_ = 5.5 × 10^–5^) for upregulated DEGs and NFE2L2 for downregulated DEGs (*P*_adj_ = 7.6 × 10^–4^). *MYC* and *E2F4* were also themselves differentially expressed in SSc compared with HCs in superficial cells (*P*_adj_ = 7.8 × 10^–5^ and *P*_adj_ = 9.8 × 10^–4^, respectively). MYC was the most enriched transcription factor in genes upregulated in GERD compared with HCs, but the enrichment was not significant after adjustment for multiple testing (*P*_adj_ = 0.11). No transcription factors were significantly enriched in any pairwise comparison in the distal esophagus.

### Fewer metallothioneins and metallothionein-expressing cells in superficial EECs in SSc.

**Figure 4:**
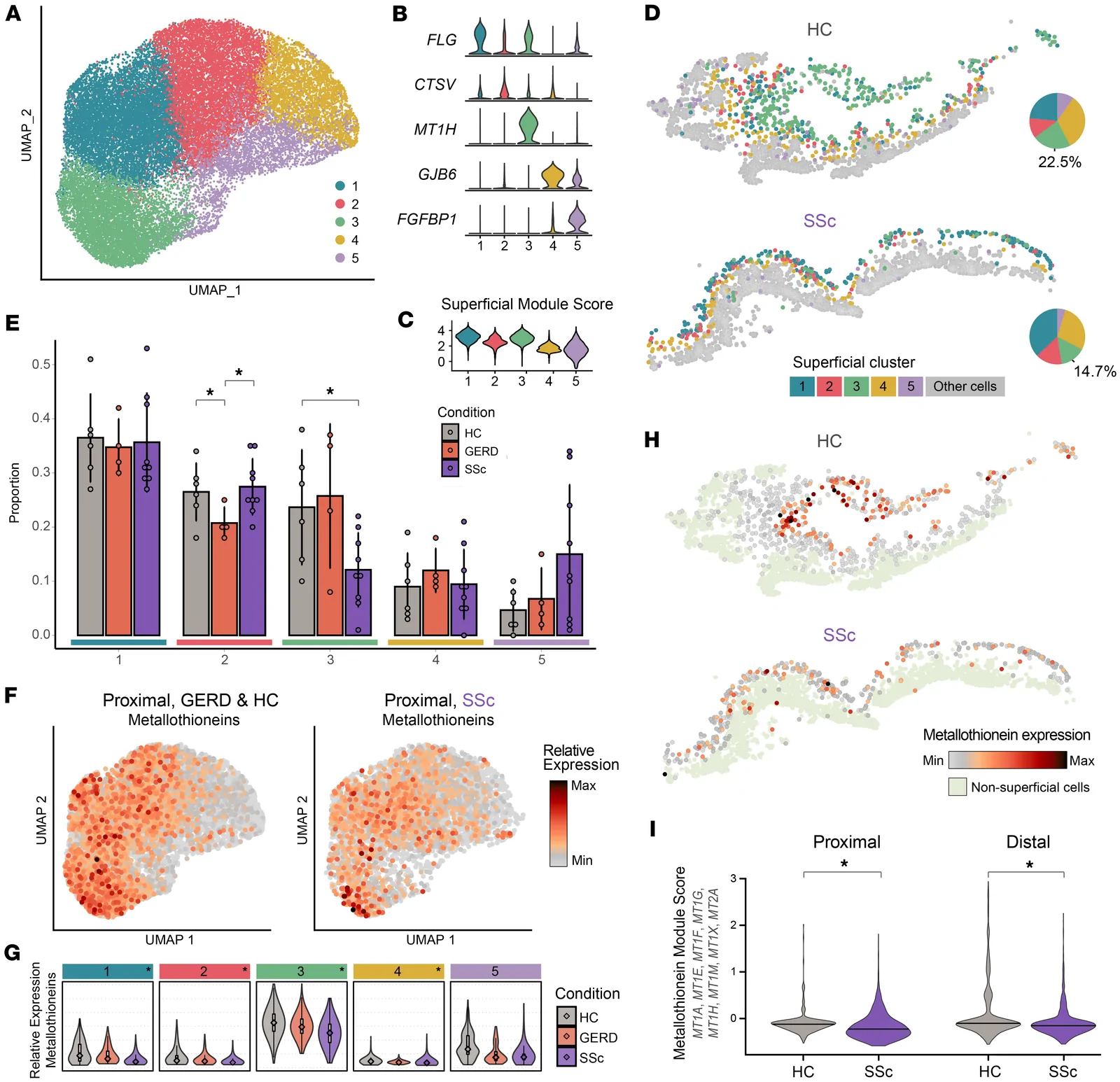
Landscape of superficial EECs. (**A**) Integrated UMAP embedding of all superficial EECs (*n* = 30,866) clustered by unique transcriptional signatures. (**B**) Relative expression of cluster-specific gene signatures. (**C**) Superficial module score by superficial cell cluster. (**D**) CosMx spatial molecular imaging of esophageal mucosal tissue from a healthy control donor and in SSc with absent contractility, annotated using label transfer from the scRNA-seq data. Superficial cluster proportions were derived from all imaged proximal tissue (*n* = 2 SSc, 2 HCs). (**E**) Proportion of superficial cells by condition and cluster from the proximal esophagus. The bars denote the mean values, the vertical lines the standard deviations, and the points the individual sample proportions. Pairwise differences across conditions were evaluated statistically using MASC (96). *P < 0.05. (**F**) Distribution of metallothionein module score in proximal, superficial cells by condition. The metallothionein module score included *MT1A*, *MT1E*, *MT1F*, *MT1G*, *MT1H*, *MT1M*, *MT1X*, and *MT2A*. (**G**) Distribution of relative metallothionein expression by cluster and condition in superficial cells from proximal esophagus. All pairwise comparisons between SSc and HCs were statistically significant, except for cluster 5. (**H**) CosMx spatial molecular imaging of esophageal mucosal tissue from a healthy control donor and in scleroderma with absent contractility. Metallothionein module scores are plotted within the superficial EECs. (**I**) Distributions of metallothionein module scores by cell cluster and condition from the CosMx spatial molecular imaging data. Significant pairwise differences (Wilcoxon’s rank-sum) in module score levels (*P*_FDR_ < 0.05) are denoted with an asterisk.

With SSc-specific dysregulation most prominent in superficial EECs, we performed subclustering of these cells to further examine corresponding expression patterns. We identified 5 distinct clusters (Figure 4A and Supplemental Figure 6A) with unique expression marker patterns (Figure 4B, Supplemental Figure 6B, and Supplemental Table 13) and varying degrees of differentiation (Figure 4C). We then performed label transfer onto the spatially mapped cells to confirm the physical distribution of these clusters within the esophageal epithelium. Clusters 1 and 3 represented the apical, terminally differentiated epithelial layers, as evidenced by their *FLG* expression (35–37) and spatial localization (Figure 4D), but cluster 3 had uniquely high expression of metallothioneins (Figure 4B and Supplemental Figure 6B). Cluster 2 was distinguished by its relatively high *CTSV* expression, while clusters 4 and 5 were the least differentiated of the superficial cells and were distinguished by their relative expression of *GJB6* and *FGFBP1*, respectively. Among proximal, superficial EECs, cluster 3 was less abundant in SSc compared with HCs with *P* less than 0.05, while no other clusters demonstrated significantly different abundances between SSc and HCs (Figure 4E). Metallothionein expression was also significantly lower in SSc compared with HCs or GERD in each cluster, except for cluster 5 (*P* < 5 × 10^–14^; Figure 4, F and G). These observations were reproduced in the spatial imaging data, where cluster 3 was less abundant in SSc samples (14.7%) than in HCs (22.5%; Figure 4D) and metallothionein expression was likewise significantly lower in superficial SSc cells compared with HCs, with a greater median difference observed in proximal cells (*P*_prox_ = 2.2 × 10^–19^, *P*_dist_ = 6.5 × 10^–18^) (Figure 4, H and I).

We further performed immunohistochemistry imaging for filaggrin (*FLG*) and metallothioneins MT1A and MT2A in proximal and distal samples from 4 HCs, 5 SSc patients, and 2 patients with non-SSc GERD (Supplemental Figure 7). Immunostaining confirmed that protein expression localization for these genes was consistent with the transcriptomic data. Relative proportions of FLG and metallothionein-expressing EECs between conditions were also directionally consistent. The proportion of FLG-expressing cells was lower in SSc compared with HCs (*P* = 0.06), and metallothionein expression was lower in proximal SSc cells (MT1A, *P* = 0.8; MT2A, *P* = 0.6), but these differences were not statistically significant.

Regarding the expression of the significantly enriched transcription factors (IRF1, MYC, E2F4, and NFE2L2; Supplemental Table 12) and their target genes, we observed several patterns (Supplemental Figure 6, C–F). For *MYC*, *E2F4*, and *NFE2L2*, each gene’s expression was highest on average in cluster 5, the least differentiated superficial cluster (Figure 4C), and by condition was highest in SSc, followed by GERD. Aggregate expression of target genes was positively correlated with transcription factor expression for *MYC* (*r* = 0.42) and *E2F4* (*r* = 0.24) and negatively correlated for *IRF1* (*r* = –0.20) and *NFE2L2* (*r* = –0.18). Correspondingly, cluster 5 featured the highest target gene expression for *MYC* and *E2F4* and the lowest for *NFE2L2*, and target gene expression differences relative to HCs were likewise correlated in SSc and GERD but were more pronounced in SSc. Notably, several of the immune-related genes uniquely upregulated in SSc (Figure 3D and Supplemental Figure 5H) displayed a similar expression pattern within superficial cells to that of *MYC* and *E2F4*, with expression unique to cluster 5 (Supplemental Figure 6G and Supplemental Table 13). For *IRF1*, however, expression of the transcription factor and its target genes differed more in GERD than in SSc relative to HCs.

### Enhanced epithelial-stromal communication in SSc.

**Figure 5:**
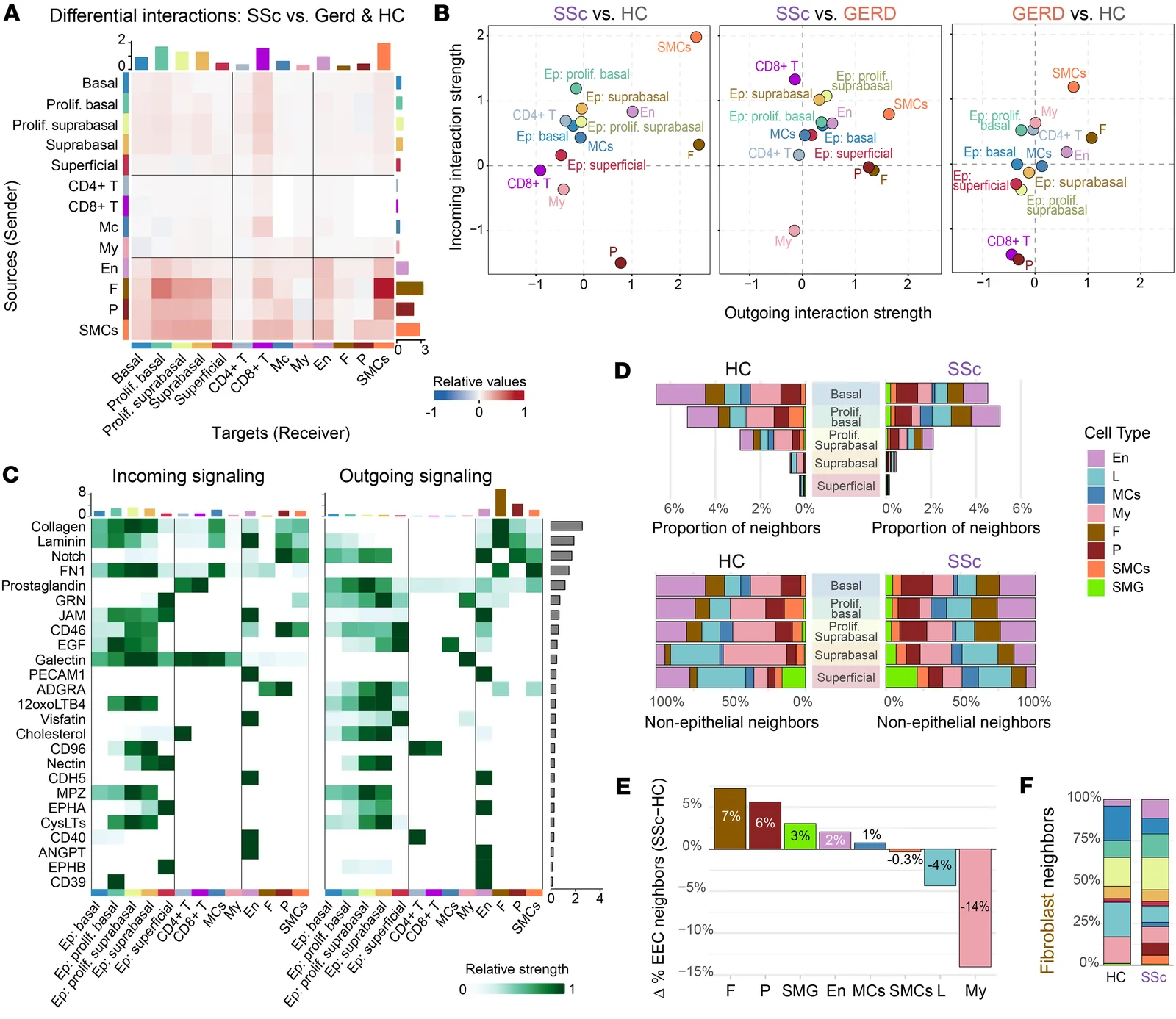
Intercellular interactions and spatial proximity. (**A**) Heatmap of differential interaction strength between SSc and combined GERD and HCs. Rows indicate source (sender) cell types, and columns indicate target (receiver) cell types. Color intensity reflects relative increase (red) or decrease (blue) in interaction strength in SSc. Marginal bar plots show aggregate differential signaling strength per cell type as senders (rows) or receivers (columns). (**B**) Differential signaling roles across pairwise condition comparisons. Each point represents a cell type, with *x* axis and *y* axis indicating differential outgoing and incoming interaction strength, respectively. Positive values indicate increased signaling in the first condition of each comparison. (**C**) Relative contribution of cell types to incoming (left) and outgoing (right) signaling for pathways with significant differential activity in SSc. Heatmap values are row-scaled. Gray bars indicate aggregate signaling strength per pathway summed across all cell types. Top colored bars indicate aggregate signaling strength per cell type summed across displayed pathways. (**D**) Spatial neighborhood composition of epithelial subtypes from CosMx spatial molecular imaging data. Top: Proportion of non-epithelial neighbors by cell type for each epithelial subtype in HC and SSc. Bottom: Relative composition of non-epithelial neighbors. (**E**) Change in the proportion of epithelial cell neighbors by non-epithelial cell type between SSc and HCs. (**F**) Cell type composition of fibroblast spatial neighbors in HCs and SSc.

To investigate whether the transcriptional changes observed in EECs were accompanied by altered intercellular communication, we applied CellChat to infer ligand-receptor interactions across cell types within each condition. We first examined global differences in cell-cell communication by quantifying the number and strength of predicted interactions between cell type pairs. Compared with HCs and GERD, SSc exhibited notably increased interactions with EECs originating from stromal cells (Figure 5A). Stromal cells demonstrated the largest increases in outgoing signaling overall in SSc, particularly from fibroblasts and smooth muscle cells (Figure 5, A and B), with the largest relative shift in predicted interactions being increased communication from fibroblasts to smooth muscle cells.

We next compared the relative activity of specific intercellular signaling pathways within SSc and between conditions using information flow analysis, which aggregates predicted signaling activity across all cell type pairs for each pathway. Among pathways with significant intercellular signaling in SSc (*n* = 63; Supplemental Figure 8A), 25 were significantly more invoked in SSc compared with both GERD and HCs (Figure 5C and Supplemental Figure 8, B and C). These SSc-enriched communication pathways included canonical signaling pathways in SSc pathogenesis such as collagen, laminin, and fibronectin (FN1) signaling from fibroblasts to EECs; Notch signaling from EECs to fibroblasts; galectin signaling from myeloid cells; and CD40 signaling from CD4^+^ T cells to endothelial cells.

To assess whether the inferred ligand-receptor interactions were histologically supported, we performed spatial proximity analysis using the spatial imaging data. We quantified the neighborhood composition of each cell type by aggregating the 5 nearest spatial neighbors within 50 μm for each cell. The vast majority of each cell type’s neighborhood consisted of other cells of that same cell type (Supplemental Figure 8D). However, for EECs, the greatest proportional increase of non-epithelial neighbors in SSc relative to HCs was with fibroblasts (+7%), and the greatest proportional decrease was with myeloid cells (–14%) (Figure 5, D and E), consistent with the predicted intercellular interactions in SSc (Figure 5, A–C). Fibroblasts in SSc demonstrated an increased proportion of stromal neighbors, while overall epithelial and immune cell proximity was proportionally reduced (Figure 5F). Together, these results indicate that the enhanced epithelial-fibroblast signaling predicted by ligand-receptor analysis is supported by increased spatial proximity between these cell populations in SSc tissue, consistent with a model in which stromal-epithelial crosstalk contributes to the fibrotic remodeling of the SSc esophagus.

### FLI1 was not expressed in human EECs.

Deletion of the *Fli1* gene in epithelial cells in mice has been shown to recapitulate histological and molecular features of esophageal involvement in SSc (15). Therefore, we investigated the expression of *FLI1* in our human esophageal samples. The expression of *FLI1* in human EECs was negligible and did not meet the minimum expression thresholds for inclusion in our differential gene expression analysis. We did observe low *FLI1* expression levels in endothelial cells (Supplemental Figure 9A), but the expression differences between SSc, HCs, and GERD were not significant (Supplemental Figure 9B). There were many FLI1 downstream targets among the proximal, superficial DEGs between SSc and HCs and seven between SSc and GERD (*ARPC2*, *RAB24*, *MTPN*, and *PPIF* upregulated; *CEBPZ*, *TRMT10C*, and *TBP* downregulated); however, the proportion of these genes among all DEGs was not different from what would be expected by chance (*P* > 0.05), and the average expression of FLI1 targets was higher in GERD and lower in HCs compared with the expression in SSc (Supplemental Figure 9C).

### Reduction of outer EECs and metallothionein expression correlates with loss of contractility in SSc.

**Figure 6:**
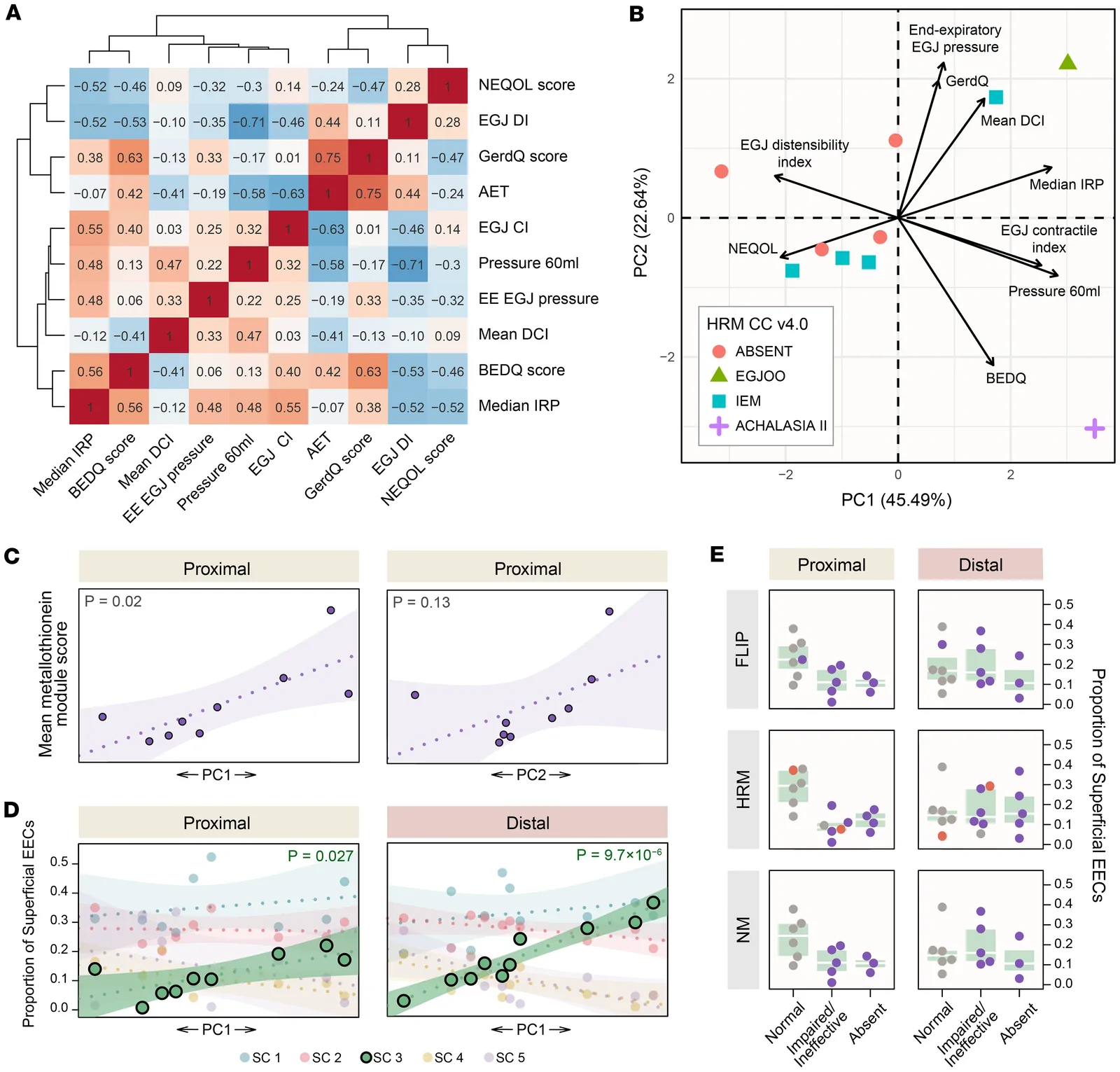
Correlations with clinical phenotypes. (**A**) Heatmap of quantitative clinical traits organized using agglomerative, complete hierarchical clustering on Euclidean distances, with pairwise Spearman correlations shown in each cell. (**B**) SSc cases are plotted on the first 2 PCs of the quantitative clinical traits, colored by esophageal motility phenotype. The relative magnitude and direction of trait correlations with the PCs are shown with black arrows. (**C**) Correlations between aggregate, relative metallothionein expression and clinical trait PCs in superficial cells in the proximal esophagus. Trendlines with 95% confidence intervals are shown with the unadjusted correlation *P* value. The metallothionein module score included *MT1A*, *MT1E*, *MT1F*, *MT1G*, *MT1H*, *MT1M*, *MT1X*, and *MT2A*. (**D**) Correlations between superficial clusters (SCs) and clinical trait PC1 in SSc cases. Linear regression trendlines with 95% confidence intervals are shown for each SC. The displayed *P* values correspond to the highlighted correlations between SC3 and PC1. (**E**) Box plots showing the proportion of SC3 in superficial cells by esophageal motility phenotype in the proximal and distal esophagus, colored by disease state (HCs, gray; GERD, orange; SSc, purple). AET, acid exposure time; BEDQ, brief esophageal dysphagia questionnaire; DCI, distal contractile integral; DI, distensibility index; EGJ, esophagogastric junction; EGJOO, esophagogastric junction outflow obstruction; FLIP, functional luminal imaging probe panometry; HRM, high-resolution manometry; IEM, ineffective esophageal motility; IRP, integrated relaxation pressure; NEQOL, Northwestern Esophageal Quality of Life; NM, neuromyogenic model.

We next evaluated whether the cellular and molecular changes we observed between SSc, GERD, and HCs were correlated with clinical measures of esophageal dysfunction (Table 1 and Supplemental Table 14). Specifically, we examined 9 quantitative measures spanning 3 domains: (a) esophageal contractility and motility (high-resolution manometry [HRM] mean distal contractile integral, which quantifies contraction strength; functional luminal imaging probe panometry [FLIP] intra-balloon pressure at 60 mL, which reflects resistance to distension), (b) lower esophageal sphincter function (esophagogastric junction [EGJ] distensibility index, basal EGJ pressure, EGJ contractile index, integrated relaxation pressure [IRP]), and (c) patient-reported symptom severity (brief esophageal dysphagia questionnaire [BEDQ] for dysphagia, GerdQ for reflux symptoms, Northwestern Esophageal Quality of Life for quality of life). To more efficiently model the covariance structure of esophageal function in SSc (Figure 6A), we performed principal component analysis (PCA) on these 9 clinical metrics and then tested for correlations with the first 2 PCs (Figure 6B). The first PC explained 45.5% of variance and was most strongly correlated with FLIP intra-balloon pressure (*r* = 0.94). PC1 effectively distinguished patients by distensibility and contractility: higher PC1 values corresponded to stronger contractility and higher pressures, while lower PC1 values indicated weaker contractility and greater EGJ distensibility. Accordingly, SSc patients classified with absent contractility clustered at low PC1 values (Figure 6B). The second PC explained 22.6% of variance and was most correlated with HRM basal EGJ pressure (*r* = 0.74), capturing additional variation in sphincter tone and distinguishing EGJ outflow obstruction from achalasia.

We did not observe any significant associations between epithelial cell compartment proportions in SSc and esophageal motility phenotypes. It appeared that the low proportion of superficial cells in the esophageal epithelium was a universal feature in SSc, regardless of phenotype (Supplemental Figure 10, A and B). The proportion of superficial cells decreased with disease duration, but the trend was not statistically significant (*P* = 0.1; Supplemental Figure 10C). We also did not observe any significant correlations between EEC compartment proportions and the quantitative trait PCs (Supplemental Figure 10D).

We then tested for correlations with gene expression in superficial EECs for genes that were significantly differentially expressed in SSc compared with HCs (*P*_adj_ < 0.05) and nominally differentially expressed in SSc compared with GERD (*P* < 0.05). There were 433 genes that met these criteria in the proximal esophagus and 99 in the distal esophagus. Although no associations with esophageal motility phenotypes were statistically significant after adjustment for multiple testing, the strongest phenotypic correlation was with *TRIM11* (*P*_FLIP_ = 0.01, *P*_HRM_ = 0.007; Supplemental Figure 10E), a gene recently found to attenuate Treg cell differentiation in CD4^+^ T cells in mice (38). No associations with the top 2 clinical PCs were significant after adjustment for multiple testing. We also assessed clinical PC correlations with aggregate metallothionein gene expression and transcription factor target expression for IRF1, MYC, E2F4, and NFE2L2. We observed one nominal association between the mean metallothionein module score and PC1 (*P* = 0.02; Figure 6C). Notably, within superficial cells we also observed a strong correlation between the proportion of metallothionein-expressing cluster 3 cells and PC1 in the proximal (*P* = 0.027) and distal esophagus (*P* = 9.7 × 10^–6^) (Figure 6D). This indicates that not only is this population of cells reduced in SSc, but its relative decrease is further correlated with more severe esophageal involvement (Figure 6E).

## Discussion

Esophageal dysfunction is extremely common in SSc and is significantly associated with increased mortality and lower quality of life (39, 40). The causal mechanisms by which SSc affects the esophagus, however, have not been conclusively determined. Here, we sought to clarify the role of epithelial cells in SSc esophageal dysfunction by using scRNA-seq to quantify cellular and transcriptional changes relative to HCs and individuals with GERD. While our findings suggest that epithelial changes in SSc result primarily from chronic acid exposure, they also highlight immunoregulatory pathways uniquely altered in SSc that may be linked to pathogenic aberrations.

The pathogenic role of epithelial cells in SSc has been an ongoing area of research (13). When damaged, epithelial cells release signals that help induce fibroblast activation to promote wound healing (41). In SSc, epidermal keratinocyte characteristics resemble a profibrotic, activated state (42). SSc epidermal cells were also found to stimulate fibroblasts in culture (43), and some signs related to epithelial-mesenchymal transition have been observed in the SSc epidermis (44). The FLI1 (friend leukemia integration 1) transcription factor, in particular, has received attention for its potential role in epithelial cell–mediated SSc pathogenesis (13). Lower FLI1 expression was observed in the epidermis of diffuse cutaneous SSc, and inactivation of FLI1 in human keratinocytes in vitro induced gene expression changes characteristic of SSc (15). Furthermore, conditional deletion of *Fli1* in epithelial cells produced an SSc-like phenotype in mice (15). Importantly, the epithelial cell–specific *Fli1* knockout further recapitulated the esophageal involvement of SSc, with increased collagen deposition in the lamina propria combined with atrophy of the circular muscle layer, and the esophageal changes were not mediated by T cell autoimmunity (15). In contrast to these previous studies in mice and epidermal keratinocytes, we did not observe *FLI1* expression in EECs in any condition. *FLI1* was expressed at low levels in endothelial cells, but the expression differences between SSc, HCs, and GERD were not significant. We did observe significantly increased expression in SSc and GERD of the *PI3* gene, which encodes trappin-2/elafin, previously found to be induced by Fli1 silencing in human dermal microvascular endothelial cells (45). However, this association is unlikely to be pathogenic, as the increase in gene expression was greater in GERD, and a growing body of evidence indicates that trappin-2/elafin is expressed to promote tissue repair in response to gastrointestinal tissue inflammation (46).

While there have been many studies of epithelial cells in skin in SSc, molecular study of the human esophagus in SSc has heretofore been limited to one array-based gene expression study in bulk tissue conducted by Taroni and colleagues (7). In their study of 15 individuals with SSc, they identified distinct expression signatures among genes with low intra-patient and high inter-patient variance that were similar to signatures seen in SSc skin (47). These signatures were independent of clinically defined SSc subsets, suggestive of inter-individual heterogeneity in SSc (7). We evaluated the expression of these signatures in our single-cell data and found that they correlated strongly with specific cell type proportions. We further found that cell type composition itself was significantly more correlated within patients across biopsy locations than between patients. Our findings suggest that the molecular subsets previously identified in bulk esophageal tissue largely reflect differential cell type composition between patients rather than distinct transcriptional programs within the same cell types. Taroni and colleagues did not observe statistical associations between inflammatory gene expression signatures and GERD, but the study was underpowered for such an analysis, and GERD was defined based on histological evidence for basal cell hyperplasia and intraepithelial lymphocyte counts (7).

The cellular composition and gene expression differences we observed among SSc epithelial cells were highly correlated with those seen in GERD, and nearly all differential gene expression was limited to the superficial layers of the epithelium. These observations suggest that the primary driver of differential gene expression in EECs in SSc is chronic acid exposure. This stands in contrast to eosinophilic esophagitis, where single-cell analysis of the esophageal epithelium revealed substantial expansion of the suprabasal compartment and no correlation with GERD gene expression signatures (19). The genes with the strongest mutual upregulation in SSc and GERD were primarily related to keratinization/cornification, including small proline-rich proteins (SPRRs), serine protease inhibitors (serpins), S100 proteins, late cornified envelope (LCE) genes, and keratins associated with terminal epithelial differentiation, namely *KRTDAP* and *KRT*. *KRT1* helps maintain epithelial barrier function in gastrointestinal epithelial cells, and its overexpression was shown to attenuate IL-1β–induced epithelial permeability (48). The mutual upregulation of these genes in the outer layers of the epithelium therefore likely represent a protective response to chronic acid exposure.

While the overall gene expression differences were highly correlated between SSc and GERD, there were sets of genes disproportionately upregulated in SSc that hint at disease-related immune response aberrations, including genes that have previously been implicated in SSc pathogenesis. For example, *PTGES*, which encodes prostaglandin E synthase, was previously found to be significantly upregulated in SSc fibroblasts (49) and inflammatory non-classical monocytes (50), and Ptges-null mice were resistant to bleomycin-induced fibrosis (49). CD44 and CD74 form the receptor complex for the macrophage migration inhibitory factor (MIF), an inflammatory cytokine that promotes fibroblast migration and has been implicated in SSc pathogenesis (51). LTB4R (leukotriene B_4_ receptor; or BLT1) has been found to activate AKT/mTOR signaling, and knockdown of *LTB4R* attenuated fibrosis in murine SSc models (52). Serum heparin-binding epidermal growth factor (HBEGF) levels and fibroblast *HBEGF* expression were both significantly elevated in SSc (53). Copy number variation of *APOBEC3A* was significantly associated with SSc in a Han Chinese population (54). Finally, the SSc-specific upregulation of genes involved in antigen processing and presentation (*HLA-B*, *CD74*, *TAP1*, *PSMB8*, *PSMB9*) is suggestive of increased human leukocyte antigen (HLA) class I activity in superficial EECs in SSc. Notably, *TAP1*, *PSMB8*, and *PSMB9* are located immediately adjacent to one another in the class II region of the HLA locus, which is the genomic region with the strongest genetic associations with SSc (55), and SSc-associated HLA-B alleles have also been identified (56, 57).

The most striking set of genes that were uniquely downregulated in SSc were metallothioneins. The expression of detected metallothioneins (*MT1A*, *MT1E*, *MT1F*, *MT1G*, *MT1H*, *MT1M*, *MT1X*, *MT2A*) was strongly reduced in the superficial compartment of EECs in the proximal esophagus in SSc compared with HCs. We further observed a nominal association between relative metallothionein expression in the proximal esophagus and the first PC of esophageal dysmotility among SSc patients, with lower expression of metallothioneins correlated with greater EGJ distensibility and weaker contractility. The proportion of the primary metallothionein-expressing cluster within superficial EECs was likewise significantly associated with clinical PC1. The metallothioneins are a family of well-conserved, metal-binding proteins that regulate zinc and copper homeostasis, prevent heavy metal poisoning, and combat oxidative stress (58, 59). Their study in a wide array of physiological conditions and immune-mediated diseases has revealed that the proteins play central roles in innate and adaptive immunity, including autoimmunity, but their immunoregulatory behavior is highly complex and context-specific (58, 59). Given their import in immune regulation, their associations with SSc and esophageal motility reported in this study, and the connection between SSc and heavy metal exposure (60), the metallothioneins are compelling candidates that warrant further study in SSc.

Targets for several transcription factors, including IRF1, MYC, E2F4, and NFE2L2 (or NRF2), were significantly enriched among DEGs in superficial EECs from the proximal esophagus. IRF1 is involved in innate immune responses, but while IRF1 targets were enriched in DEGs between SSc and GERD, the enrichment appeared to be driven by upregulation in GERD. IRF1 targets were not enriched in DEGs between SSc and HCs, nor was *IRF1* significantly differentially expressed in SSc. MYC, E2F4, and NFE2L2 all participate in different stages of the cell cycle (61–63), and expression of each was highest in either the proliferating basal or suprabasal compartments, but these proteins may also play a role in terminal differentiation of EECs, particularly under conditions of stress. In the least differentiated superficial cluster (cluster 5), where the expression differences relative to HCs for each of these transcription factors and their targets were most pronounced, the expression level changes in GERD were correlated with those observed in SSc. Furthermore, Myc ablation in Krt14-expressing tissues in mice led to abnormal terminal differentiation of epithelial cells (64), and studies of the esophageal epithelium in Nrf2-knockout mice indicate that Nrf2 regulates cornification of keratinized epithelial cells under oxidative stress (65, 66). Moreover, *C10orf99* — one of the genes uniquely upregulated in SSc with superficial expression mirroring *MYC*, *E2F4*, and *NFE2L2* — was found to increase expression of proinflammatory markers and attenuate late differentiation of keratinocytes under conditions of stress (67). These findings therefore suggest that within the SSc esophagus there is activation of pathways that regulate the late differentiation of EECs in response to external stress, and these could contribute to the reduction of superficial EECs that we observed in SSc.

Together, the SSc-specific downregulation of metallothioneins and NRF2 targets and upregulation of immune mediators such as *PTGES*, *LTB4R*, *CD44*/*CD74*, and *HLA* class I pathway genes in superficial EECs highlight pathways that have been explored as therapeutic targets in SSc and related fibrotic diseases. Preclinical studies support a pathogenic role for oxidative stress in SSc and suggest that pharmacological activation of NRF2 or augmentation of antioxidant defenses can attenuate fibrosis and inflammation (68, 69). Increasing of metallothionein expression through zinc supplementation has been shown to reduce fibrosis in experimental models of other fibrotic diseases (70–74). Zinc deficiency is common in SSc (75), and although studies of zinc supplementation in SSc are limited (76), there is evidential support for zinc supplementation as a potential adjunct therapy in SSc (77). Vipoglanstat, an mPGES-1 (PTGES) inhibitor, received FDA orphan drug designation for SSc, but failed to reduce SSc-associated Raynaud’s phenomenon more than placebo in a phase II trial (78). Several classes of MIF inhibitors have been in clinical development for other autoimmune diseases (79), including rheumatoid arthritis and systemic lupus erythematosus, but have not yet been examined for SSc. LTB4R has been investigated as a therapeutic target for immune diseases, but LTB4R antagonists have so far failed in phase II testing (80). Our data suggest that these strategies may also impact epithelial compartments, in which impaired antioxidant defenses and heightened immune activation could contribute to ongoing tissue injury in the SSc esophagus.

Interestingly, there was greater relative gene dysregulation in the proximal esophagus compared with the distal esophagus in SSc, but the opposite was true for GERD. This discrepancy suggests that esophageal dysmotility in SSc leads to greater acid exposure in the proximal esophagus, since refluxate typically affects the distal esophagus significantly more than the proximal esophagus (81) and abnormal peristalsis prolongs acid clearance (82). This finding coincides with a bulk RNA-seq study of esophageal mucosa of achalasia patients (83), which likewise identified more differential expression in the proximal esophagus than in the distal esophagus, including significant enrichment of matrisome-associated genes and lower expression of genes associated with reactive oxygen species metabolism (83). Because of differences in innervation depth (84), the epithelium of the proximal esophagus has more nociceptive sensitivity than the distal esophagus, which likely contributes to the association between gastrointestinal symptoms and lower health-related quality of life in SSc (85). Proximal acid exposure increases the risk of aspiration (86) and is significantly more prevalent in SSc patients with idiopathic lung fibrosis (87).

The transcriptional changes observed in SSc EECs were accompanied by altered intercellular communication in the tissue microenvironment. By examining predicted ligand-receptor interactions across cell types, we found that fibroblasts in SSc demonstrated markedly increased outgoing signaling to smooth muscle cells and EECs via collagen, laminin, and fibronectin (FN1) pathways. Conversely, there was greater predicted signaling from EECs to fibroblasts via the Notch pathway. This predicted increase in profibrotic signaling was supported by greater observed spatial proximity between fibroblasts and epithelial cells in the spatial transcriptomics data. These profibrotic signaling axes are central to SSc pathogenesis (88, 89) and are consistent with the smooth muscle atrophy and collagen deposition that characterize esophageal involvement in SSc (1). Notably, this enhanced stromal signaling was much more evident in SSc than in GERD, further distinguishing the otherwise largely shared epithelial transcriptional response between the two conditions. The SSc epithelium thus exists within a microenvironment of chronic acid exposure combined with heightened profibrotic stromal signaling that may compound epithelial injury. Damaged epithelial cells can release signals that further activate fibroblasts, potentially establishing a self-reinforcing cycle of epithelial injury and fibrotic remodeling in the SSc esophagus.

There are important limitations to consider when interpreting this study. First, this study primarily focused on squamous epithelial cells. There may be relevant changes in other cell types within the esophageal mucosa that correlate with SSc and dysmotility, but such analyses are ongoing and were outside the scope of this investigation. Furthermore, endoscopic biopsies from deeper tissue layers, such as the muscularis propria, are infeasible (90). Second, observed differences in cell type proportions between conditions could have been influenced by condition-associated variation in cell recovery during tissue dissociation, as disease-related changes in tissue architecture or cell fragility may affect how efficiently certain cell types are isolated. Third, the relatively small patient sample size of this study combined with the heterogeneity of SSc subtypes and dysmotility phenotypes limited our power to detect clinical associations between cell type proportions or gene expression changes and specific clinical phenotypes. Heterogeneous molecular profiles in SSc esophagus samples have been described previously (7), but we grouped all SSc patients together in our analyses for comparisons against GERD and HCs. We applied ordinal logistic regression on motility classifications and performed PCA on reflux- and motility-related quantitative traits to increase our power to detect clinical associations but were nonetheless statistically limited by sample size. Fourth, the HC samples used in this study were collected from individuals who were significantly younger than the SSc and non-SSc GERD patients. Previous scRNA-seq of esophageal epithelium in mice identified age-associated transcriptional changes in pathways related to mitochondrial function and oxidative stress, although epithelial cell composition did not change with age (91). In humans, age is associated with increased reflux prevalence (92), and among those with reflux symptoms, age is associated with higher acid exposure and lower esophageal motility (93). Therefore, age differences between conditions in this study could have contributed to differential gene expression signals. Fifth, proton pump inhibitor (PPI) use differed between SSc (80%) and non-SSc GERD (25%) patients, reflecting standard clinical management of SSc-associated esophageal disease. However, the high correlation between SSc and GERD transcriptional signatures relative to HCs suggests that shared disease biology, rather than differential PPI exposure, drove the observed gene expression changes. Additionally, prior in vivo studies have shown that PPI therapy normalizes esophageal gene expression toward that in healthy controls rather than inducing distinct transcriptional signatures (94, 95). Sixth, our analysis of spatial transcriptomic data in the SSc esophageal mucosa was limited. While the spatial molecular imaging confirmed our cell annotations and reproduced findings from scRNA-seq, the CosMx cell segmentation algorithm could not aptly capture the flattened, densely packed outer cell layers of the epithelium, resulting in a relatively sparse representation of superficial EECs. Furthermore, the spatial analysis was limited to 8 samples from 4 individuals. Therefore, we cannot make more robust conclusions on the morphological states that accompany the transcriptional and cellular proportion changes that we observed. Future studies using larger, more homogeneous cohorts with complete imaging data may be able to detect more esophageal epithelial changes associated with dysmotility in SSc.

In summary, esophageal complications are common in individuals with SSc and can greatly impact quality of life and lifespan. In this study, we sought to deepen our understanding of the role of the epithelium in SSc esophageal involvement. Through a thorough, single-cell-level transcriptomic investigation, we identified the unique cellular and transcriptional differences present in the SSc esophageal epithelium. There were significantly fewer terminally differentiated epithelial cells in the apical, superficial layers in both the proximal and distal esophagus, but otherwise epithelial compartment proportions were similar to those in healthy controls, including proliferating cell proportions. Significant differences in gene expression were likewise almost exclusively found in the superficial compartment, and these changes were highly correlated with those seen in individuals with GERD, indicating that the primary driver of differential gene expression in the SSc esophageal epithelium is chronic acid exposure. Transcriptional differences were more prominent in the proximal esophagus of SSc patients versus GERD patients, possibly owing to greater proximal exposure resulting from esophageal dysmotility. Genes that were most disproportionately dysregulated in SSc compared with GERD belonged to immune-related pathways, including innate and adaptive response, antigen presentation, and metal homeostasis, pointing to pathogenic immune dysfunction. These changes coincided with significantly increased intercellular signaling with fibroblasts. By serving as an atlas for the human esophageal epithelium in SSc, this study can guide future efforts to address remaining gaps in our understanding of the SSc esophagus, to ultimately uncover pathogenic mechanisms and identify actionable targets.

## Methods

Detailed methods regarding study subjects, clinical evaluation, sample processing, imaging, and all computational analyses are provided in Supplemental Methods.

### Sex as a biological variable.

Both male and female participants were included in this study. Our cohort was predominantly female (17 of 20 participants; Table 1), consistent with the female predominance of SSc. Biological sex was not considered as an independent variable in our analyses.

### Statistics.

Statistical analyses were performed using R version 4.3.0. Descriptive statistics are displayed as medians with interquartile ranges for continuous variables unless otherwise described and as frequency counts for categorical variables. For non-normally distributed continuous data, Wilcoxon’s rank-sum tests were used. When multiple conditions were tested, multiple-testing correction was applied. Corrected *P* values less than 0.05 were considered statistically significant. For box plots, boxes denote the interquartile range (IQR), the center line the median, and whiskers extend to the most extreme values within 1.5× the IQR, unless otherwise noted.

### Study approval.

The study protocol received approval from the Northwestern University Institutional Review Board. Informed consent was obtained from all participants.

### Data availability.

Raw and filtered matrices of unique molecular index counts per gene per sample were deposited to the NCBI’s Gene Expression Omnibus database under accession GSE295274. Code used to generate the results of this study is available at https://github.com/mdapas/SSc_eso_epi_sc Values for all data points in graphs are reported in the Supporting Data Values file. All other supporting data are available within the article and/or supplemental material.

## Author contributions

MPT, JEP, and HRP conceived and designed the study. MH, JEP, DAC, MC, and KA facilitated patient recruitment and sample collection. DAC, CLR, MH, and JEP provided clinical analyses and oversight. TT managed sequencing data and executed alignment and quantification pipelines. MHC, HMM, HRP, DRW, and MPT provided biological analyses and oversight. KM facilitated immunohistochemistry analysis. LNM aided in biostatistical analysis. PSD and CW facilitated spatial transcriptomics data generation. MD performed all data analyses and data visualization and wrote the manuscript. All authors read and approved the manuscript. HRP, DRW, and MPT jointly supervised the study.

## Conflict of interest

DAC has received consulting or speaking fees from Medpace and Medtronic; LNM from the American College of Surgeons; CLR from Cabaletta Bio; PSD from Abbvie, Abivax, Adiso, Alimentiv, Bristol Meyer Squibb, Celltrion, Genentech, Genenoscopy, Janssen, Pfizer, Sanofi, and Takeda; JEP from Covidien, EndoGastric Solutions, HS Consolidated, Medtronic Logistics, Medtronic, Phathom Pharmaceuticals, UpToDate, and WebMD; HRP from the American Society for Clinical Investigation, AnaptysBio, *Arthritis Research & Therapy*, General Dynamics Information Technology, and L.E.K. Consulting; and DRW from Gerson Lehrman Group. DAC and JEP have a licensing agreement with Medtronic, and PSD with the University of California, San Diego.

## Funding support

This work is the result of NIH funding, in whole or in part, and is subject to the NIH Public Access Policy. Through acceptance of this federal funding, the NIH has been given a right to make the work publicly available in PubMed Central.

- NIH National Institute of Diabetes and Digestive and Kidney Diseases P01DK117824 (to MPT) and U01DK134321 (to PSD).
- NIH National Institute of Allergy and Infectious Diseases R01AI163742 (to DRW) and U19AI181102 (to PSD).
- NIH National Institute of Arthritis and Musculoskeletal and Skin Diseases R01AR080513 (to DRW and HRP), R01AR075423 (to HRP), and R01AR073270 (to MH).
- Digestive Health Foundation (to MPT).
- Northwestern University NUSeq Core Facility (NIH 1S10OD025120).
- Northwestern University Metabolomics Core Facility (NIH 1S10OD034357).
- Northwestern University Pathology Core Facility (NIH National Cancer Institute P30CA060553).

## Supplementary Material

Supplemental data

Supplemental tables 1-13

Supporting data values

## Figures and Tables

**Table 1:**
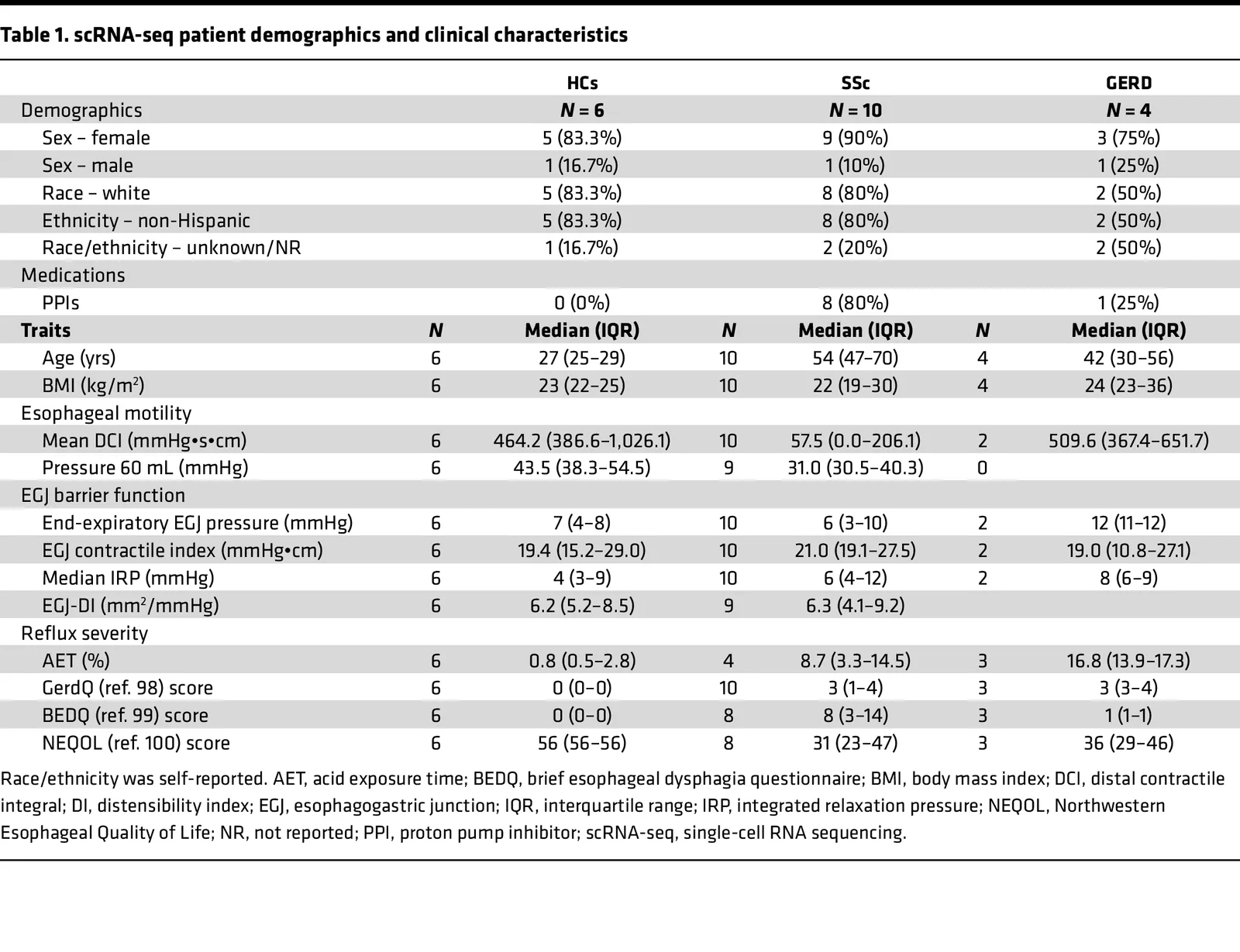
scRNA-seq patient demographics and clinical characteristics

## Version 1 07/21/2026

In-Press Preview

## Version 2 08/24/2026

Electronic publication
