# Supplementary material for: Cellular and molecular dysregulation of the esophageal epithelium in systemic sclerosis
Source: JCI Insight. 2026 Jul 16;11(16):e195322. doi: 10.1172/jci.insight.195322 (PMC13502173; doi:10.1172/jci.insight.195322)
Supplement: Supplementary file 1 [file jciinsight-11-195322-s253.pdf]

SUPPLEMENTAL MATERIAL

Cellular and molecular dysregulation of the esophageal epithelium in systemic sclerosis

Matthew Dapas[1,2]✉, Margarette H. Clevenger[3], Hadijat M. Makinde[1,2], Tyler Therron[1], Dustin A. Carlson[3,4], Mary Carns[1], Kathleen Aren[1], Cenfu Wei[3], Kainat Mian[1], Lutfiyya N. Muhammad[5], Carrie L. Richardson[1], Parambir S. Dulai[2,3], Monique Hinchcliff[6], John E. Pandolfino[3,4], Harris R. Perlman[1,2*], Deborah R. Winter[1,2*], Marie-Pier Tetreault[2,3*]

1. Division of Rheumatology, Department of Medicine, Northwestern University Feinberg School of Medicine, Chicago, IL, USA
2. Center for Human Immunobiology, Northwestern University Feinberg School of Medicine, Chicago, IL, USA
3. Division of Gastroenterology & Hepatology, Department of Medicine, Northwestern University Feinberg School of Medicine, Chicago, IL, USA
4. Kenneth C. Griffin Esophageal Center of Northwestern Medicine, Northwestern University Feinberg School of Medicine, Chicago, IL, USA
5. Department of Preventive Medicine, Northwestern University Feinberg School of Medicine, Chicago, IL, USA
6. Section of Rheumatology, Allergy & Immunology, Department of Medicine, Yale School of Medicine, New Haven, CT, USA

*These authors jointly supervised the work

✉ **Corresponding author:**

Dr. Matthew Dapas, PhD

Northwestern University Feinberg School of Medicine

240 E. Huron St.

McGaw M-327

Chicago, IL 60611

Phone: 312-503-0518

E-mail: mdapas@northwestern.edu

SUPPLEMENTAL METHODS

Subjects

The SSc study population (n=10) were recruited from adult patients aged 18 to 89 diagnosed with SSc according to the 2013 ACR/EULAR Classification Criteria, who visited the Northwestern Medicine Esophageal Center between 2020 and 2021for esophageal symptom evaluation and motility testing, as described previously (1). Patients with non-SSc GERD (n=4) were recruited at the first clinic visit following positive wireless pH testing (2). Patients with technically inadequate panometry or manometry studies, previous foregut surgeries (including prior pneumatic dilation), candida esophagitis, history of esophageal cancer, or mechanical obstructions in the esophagus such as esophageal stricture, eosinophilic esophagitis, severe reflux esophagitis (Los Angeles classification C or D), or hiatal hernias larger than 5 cm were excluded. HC samples were collected from healthy, living adults (n=6) who met asymptomatic criteria including lack of esophageal symptoms, no history of alcohol dependency or tobacco use, body mass index less than 30kg/m^2, and no current antacid or proton pump inhibitor treatment (2).

Symptom evaluation and motility testing

Esophageal function testing included functional lumen imaging probe (FLIP) panometry during sedated endoscopy, high-resolution manometry (HRM), and in some subjects, 24-hour pH-impedance or timed-barium esophagogram, which were completed and interpreted as previously described (3-6). HRM classifications were based on the application of the Chicago Classification v4.0 to 10 supine and 5 upright test swallows (6). FLIP panometry classifications were based on previous evaluation of asymptomatic volunteers and patients (7-9). During endoscopy (same encounter as FLIP), two esophageal mucosal biopsies were obtained from each patient: one at approximately 5-cm (“distal esophagus”) and one 15-cm (“proximal esophagus”) from the squamocolumnar junction using Boston Scientific Radial Jaw 4 standard capacity forceps. Acid exposure was measured as the percentage of time with esophageal pH<4 over a monitoring period of 24 hours. Most patients also completed validated symptom-questionnaires (patient-reported outcome measures) on the day of esophageal motility testing with FLIP or HRM, including the Brief Esophageal Dysphagia Questionnaire (BEDQ), the Gastroesophageal Reflux Disease Questionnaire (GerdQ), and the Northwestern esophageal quality of life (NEQOL) survey (10-12). SSc disease duration was measured from onset of the first non-Raynaud symptom.

Single-cell RNA-sequencing sample processing and sequencing

Esophageal mucosal biopsies from proximal and distal esophagus were processed for scRNA-seq as described in Clevenger et al (2). Briefly, following tissue digestion and filtering, cell suspensions met 85% minimum viability via Cellometer Auto2000 (Nexcelom Bioscience). Cells were loaded into a Chromium iX controller (10X Genomics) on a Chromium Next GEM Chip G (10X Genomics) to capture approximately 10,000 cells per sample and were processed for encapsulation following the manufacturer’s protocol. Cell barcoding and library construction were performed using the 10X Genomics Chromium Next GEM Single Cell 3’ Reagents Kits v3.1 and

Dual Index Kit TT Set A according to the manufacturer's protocol. Sequencing was performed by the NUSeq Core using the Illumina NovaSeq 6000. Paired-end reads consisting of a 28-base-pair read for cell barcodes and unique molecular identifiers and a 90-base-pair read for transcripts were aligned to the GRCh38 reference transcriptome using Cell Ranger v6.1.2.

Single-cell sample and cell quality control

Samples with high ambient RNA (<70% of sequencing reads mapped to cells) were removed from consideration. Gene expression counts generated by Cell Ranger were analyzed using Seurat v4.3.0 (13) in R v4.3.0. Cells with low gene diversity (<200 unique genes), low unique molecular identifier counts (<500), or excessive counts (>100,000) were removed from consideration. Cells with high proportions of mitochondrial DNA reads, indicative of apoptosis or lysis, were removed using the bivariate regression models implemented in the miQC package (14). Cells were filtered if their proportion of reads mapped to mitochondrial DNA exceeded 0.05 and their posterior probability of belonging to a compromised cell distribution was greater than 0.75 according to miQC. Doublets, in which more than one cell is clumped together in a single droplet, were predicted and removed using scDblFinder (15) with an expected, additive doublet rate of 0.01 per thousand cells with standard deviation of 0.01.

Sample integration and cell type annotation

Individual scRNA-seq expression profiles were normalized and integrated on 3,000 variable features using Seurat's SCTransform procedure (16) with v2 regularization (17) and canonical correlation analysis dimension reduction. Integration anchors were determined using a reference consisting of three pairs of proximal and distal samples from HCs. Principal component (PC) analysis was performed on the transformed and integrated expression counts. Uniform manifold approximation and projection (UMAP) (18) embeddings were calculated on the top 40 PCs with n=200 neighbors. Cell clustering was performed using Seurat's modularity-based clustering on a shared nearest neighbor (SNN) graph with k=25 on the top 40 PCs. Clusters were annotated according to the expression of established cell-specific gene markers (**Supplemental Figure 1**). For annotation of smaller cell clusters, we first filtered out the epithelial, myeloid, lymphoid, and endothelial cell clusters and then recomputed PCs and re-clustered the subset (PCs = 25, k=15). Pairwise Pearson correlations of cell type proportion vectors were computed within patients (proximal vs. distal) and between all other sample pairs, and compared by Wilcoxon rank-sum test.

Intrinsic subset analysis

Gene annotations for differentially expressed genes between intrinsic subsets were collected (Additional file 11 from Taroni et al. (19)) and updated to current HUGO Gene Nomenclature Committee nomenclature using the HGNChelper R package (20). From the original probe list, we identified 5,261 subset-associated genes, from which 4,999 (95%) were mapped to genes in our single-cell data. For each subset expression signature (Inflammatory: upregulated, Inflammatory: downregulated, Proliferative, and Non-inflammatory), composite singe-cell scores were derived using Seurat's AddModuleScore() function. Spearman correlations were computed for each sample

between the mean module scores and the proportions of each cell type. Pseudobulk expression counts were aggregated across all cells and TMM normalization was applied via calcNormFactors from edgeR v3.42.4 (21). Genes with log counts per million (CPM) >1 in fewer than 50% of samples were excluded (n=145). Hierarchical clustering of samples was then performed on 4,854 Z-standardized, signature-associated genes using Pearson correlation distance and average linkage (as in Taroni et al. (19)).

Epithelial cell characterization

After isolating the subset of epithelial cell clusters, we further filtered cells with disproportionately low read counts (≤3,500; **Supplemental Figure 3, A-E**). The samples were then re-integrated using the procedure described above. UMAP embeddings were calculated on the top 35 PCs with n=100 neighbors. Epithelial compartments (basal, proliferating basal, proliferating suprabasal, suprabasal, and superficial) were identified according to expression of established compartment-specific and cell cycle markers (**Supplemental Figure 3F**) (2, 22-25). We computed corresponding composite gene signature scores using Seurat's AddModuleScore() function for the basal (*PDPN*, *IGFBP3*, *COL17A1*, *KRT15*, *DST*), suprabasal (*KRT4*, *KRT13*, *SERPINB3*, *GBP6*, *IVL*), and superficial (*KRT17*, *KRT78*, *FLG*, *CNFN*, *CRCT1*) compartments (**Supplemental Figure 3G**). Cells were initially clustered on an SNN graph with k=50 with resolution=0.3 (**Supplemental Figure 3, H-I**), but because the modularity optimization clustering implemented in Seurat grouped proliferating cells across different epithelial compartments and inconsistently distinguished suprabasal and superficial cells, we applied K-means clustering on the expression of the compartment-specific markers (**Supplemental Figure 3F**). We performed separate K=2 K-means clustering on 1) the Seurat clusters containing basal and early suprabasal cells (clusters 2, 5-8) to distinguish basal from suprabasal compartments and 2) the Seurat clusters containing late suprabasal and superficial cells (clusters 1, 3, 4) to demarcate suprabasal and superficial compartments (**Supplemental Figure 3, J-K**). Proliferating epithelial cells were identified using Seurat's CellCycleScoring() function. Cells predicted as being in S or G2/M phases based on the relative expression of corresponding markers (26) were labeled as proliferating cells (**Supplemental Figure 3L**). We tested for statistical differences in proportions of epithelial cell populations between sample groups using MASC (27).

Gene expression analysis

To identify dysregulated pathways in SSc, we first calculated differential gene expression between conditions (SSc, non-SSc GERD, HCs) within each epithelial compartment, considering each cell as a sample. Differential gene expression was calculated using the FindMarkers() function in Seurat with the Wilcoxon rank-sum test on log-normalized RNA counts for genes expressed in at least 1% of cells by compartment. We adjusted for multiple testing using a Bonferroni correction accounting for the number of genes tested. Genes with an adjusted P<0.05 and absolute $\log_2$ fold change >0.1 were considered significantly differentially expressed. (28, 29) Genes were then ranked by the magnitude and statistical certainty of their expression differences:

$$Weight = -\log_{10}(p) * abs(\log_2[FC])$$

Gene set enrichment was calculated using 3,795 canonical pathway gene sets from the Human Molecular Signatures Database (30) using gene set enrichment analysis (GSEA) (28, 29). Statistical gene set enrichment was calculated using one-way positive enrichment with 100,000 permutations on pathways with at least 10 genes. Mitochondrial and ribosomal genes were excluded from the GSEA.

For pseudobulk differential expression analysis we aggregated RNA counts per sample within epithelial compartments. Genes with at ≥1 CPM detected in at least 75% of samples in at least one condition per epithelial compartment were analyzed. To mitigate outlier-driven signals, genes with log-transformed expression greater than 3 standard deviations from the mean in exactly one individual were excluded. Inter-sample heterogeneity was assessed using principal coordinate analysis, as implemented in the limma plotMDS() function and with default parameters (31). Differential gene expression was evaluated using edgeR v3.42.4 (21, 32, 33). Gene expression was modeled using a quasi-likelihood negative binomial generalized linear model, with global negative-binomial trended dispersion estimated across all samples and gene-specific quasi-likelihood dispersions estimated within each epithelial compartment. Differences in gene expression across conditions were assessed using quasi-likelihood F-tests with false-discovery rate (FDR) adjustment. Differences in the proportions of genes that were significantly differentially expressed between groups were assessed using a chi-square test. Differences in gene expression profiles between biopsy locations were determined by measuring intra-individual correlations for the 2,000 most variable genes at the single-cell level aggregated by sample, epithelial compartment, and biopsy location.

Transcription factor enrichment analyses were conducted on the pseudobulk results using the enricher() function from clusterProfiler v4.8.3 (34). We used consensus results from ENCODE (35) and ChEA (36), as curated by the Ma'ayan Lab (37) and available at http://amp.pharm.mssm.edu/Enrichr/ (38) to test for the enrichment of transcription factor target genes from among genes that were differentially expressed between SSc, GERD, and HCs with Bonferroni-adjusted $P<0.05$. Background gene sets were comprised of the genes included in the pseudobulk differential expression analyses for each epithelial compartment. Transcription factors absent from the background gene set were removed as candidate transcription factors. Separate enrichment tests were performed for all differentially expressed genes and those that were upregulated or downregulated in SSc. Enrichment results were adjusted using a Bonferroni correction accounting for the number of transcription factors with more than 2 genes that were significantly differentially expressed.

Pseudobulk sample permutation

Because of the unequal sample sizes across disease groups (n=10 SSc, n=4 GERD), we permuted differential expression testing with equivalent sample sizes to better compare the relative extent of dysregulation by EEC compartment and biopsy location between SSc and GERD. We permuted all possible combinations of SSc samples with sample size n=4 for both the proximal and distal regions and used the median statistics in the SSc vs. HC tests to more directly compare the results with those observed in GERD vs. HCs.

Immunohistochemistry

At the time of endoscopic biopsy collection, a portion of each specimen was allocated for formalin fixation and paraffin embedding (FFPE). Proximal and distal esophageal biopsies were fixed separately. H&E and immunohistochemical staining were performed by the Robert H. Lurie Comprehensive Cancer Center Pathology Core Facility. Serial sections (4 µm) were cut, and mounted on charged glass slides. Sections underwent deparaffinization, rehydration, and antigen retrieval. Endogenous peroxidase activity was quenched, and non-specific binding was blocked with 5% normal serum. Sections were incubated with the following primary antibodies: anti-Filaggrin (FLG; clone FLG/1561, mouse monoclonal, ab218395, Abcam, 1:400 dilution), anti-MT1 (rabbit polyclonal, PA5-121778, Invitrogen, 1:25 dilution), and anti-MT2A (rabbit polyclonal, PA5-102549, Invitrogen, 1:25 dilution). Species-appropriate HRP-conjugated secondary antibodies were applied, and signal was visualized using 3,3'-diaminobenzidine (DAB) chromogen. Sections were counterstained with hematoxylin. Stained slides were digitized by whole slide imaging in NanoZoomer Digital Pathology Image (.ndpi) format and cropped using NDP.view2 software (Hamamatsu Photonics).

Whole slide images were analyzed using QuPath (v0.7.0) (39). For each slide, epithelial cells were manually traced. Regions exhibiting tissue folding, processing artifacts, or areas of non-specific DAB deposition were excluded from analysis. Image-specific stain vectors were estimated using QuPath's automatic detection with default parameters. Epithelial sections were segmented using SLIC superpixel segmentation (40) with 25 µm spacing and regularization = 0.5. For filaggrin, the maximum DAB OD per tile was used for quantification. A fixed optical density threshold was applied uniformly across all images. Tiles with maximum DAB OD > 0.3 were classified as filaggrin-expressing. For metallothionein stains (MT1A and MT2A), the mean DAB optical density (OD) per tile was used as the quantification metric, accounting for more diffuse expression. To further account for inter-slide variability in baseline staining intensity, positive tiles were identified as exceeding three times the median absolute deviation (MAD) above the median, per image. Tile-level differences in positivity between conditions were assessed using mixed-effects logistic regression; within-location tests included a random intercept for image, fitted separately for proximal and distal esophagus; and tests combining biopsy locations included location as a fixed effect and random intercepts for image and subject.

Spatial imaging sample preparation

Tissue sections were mounted on Leica Biosystems Apex BOND Superior Adhesive Slides (cat# 3800040) and subsequently baked overnight at 60 °C, followed by preparation for in-situ hybridization (ISH) by deparaffinization in CitriSolv (2x 5min), 100% ethanol (2x 5min) and heat-induced epitope retrieval (HIER) at 100 °C for 15 min using low pH citrate buffer (NanoString/Bruker). After HIER, tissue sections were digested with Proteinase K (2 µg/ml) diluted in PBS at 40 °C for 30 min. Tissue sections were then washed twice with diethyl pyrocarbonate (DEPC)-treated water (DEPC H2O) and incubated in 0.0015% fiducials (NanoString/Bruker) in 2X saline sodium citrate, 0.001% Tween-20 (SSCT solution) for 5 min at room temperature in the dark. Excess fiducials were rinsed

from the slides with 1X PBS, then tissue sections were fixed with 10% neutral buffered formalin (NBF) for 5 min at room temperature. Fixed samples were rinsed twice with Tris-glycine buffer (0.1 M glycine, 0.1 M Tris-base in DEPC H2O) and once with 1X PBS for 5 min each before blocking with 100 mM N-succinimidyl (acetylthio)acetate (NHS-acetate, Thermo Fisher Scientific) in NHS-acetate buffer (0.1 M NaP, 0.1% Tween pH 8 in DEPC H2O) for 15 min at room temperature. The sections were then rinsed with 2X saline sodium citrate (SSC) for 5 min and an Adhesive Hybridization Chamber (NanoString/Bruker) was placed over the tissue. ISH probes were prepared by incubation at 95 °C for 2 min and submerged in ice, and the ISH probe mix was pipetted into the hybridization chamber. The chamber was sealed to prevent evaporation, and hybridization was performed at 37 °C for 18 hrs. Tissue sections were washed twice in 50% formamide (Ambion) in 2X SSC at 37 °C for 25 min, washed twice with 2X SSC for 2 min at room temperature, and blocked with 100 mM NHS-acetate in the dark for 15 min. In preparation for loading onto the spatial imaging instrument, a flow cell was affixed to the slide.

Spatial molecular imaging and data processing

Single-cell spatial transcriptomic analysis was performed using the CosMx Spatial Molecular Imager (NanoString/Bruker), featuring the 6k Human Discovery Panel gene list, which consists of 6,175 RNA targets designed to provide coverage across all major biological pathways. Spatial fields of view (FOVs) with average counts per cell <100 were removed from consideration. Cells with low RNA counts (<20), high proportions of counts from negative probes (>0.1), extreme size (P<0.01 by Grubb's test), or more detected genes than counts were removed. Gene probes with expression levels below median background noise, as determined by negative probe detection, were also removed. Counts were log normalized using Seurat (13). Cell segmentation errors were predicted using scDblFinder (15). To annotate the cells, we performed label transfer from the single-cell dataset with SingleR (41) using default parameters on the intersect of quantified genes. Gene expression differences between groups in the spatial data were evaluated using Wilcoxon rank-sum tests. We conducted a spatial proximity analysis using a k-nearest neighbor approach (42). For each cell, we identified the k=5 nearest neighboring cells based on Euclidean distance between cell centroids, with a maximum distance threshold of 50 µm.

Intercellular communication analysis

Cell-cell communication was inferred using CellChat (v2.2.0) (43). For each condition, we first identified ligands and receptors from CellChat's manually curated database of literature-supported interactions in humans that were significantly overexpressed in each cell type relative to all other cell types. Statistical significance was assessed via permutation of cell type labels with 40 iterations, and interactions with P<0.05 retained for downstream analysis. Epithelial cells were down-sampled to 100,000 cells and cell types with fewer than 10 cells in a condition were excluded. Pathway-level communication probabilities were calculated by summing across all ligand-receptor pairs belonging to each pathway. Network centrality analysis was applied to identify dominant sender and receiver cell types for each signaling pathway. To detect differential pathway activity between conditions, we compared

information flow, defined as the sum of communication probabilities across all cell type pairs, using a paired Wilcoxon test.

Clinical phenotype associations

To test for associations with categorical measures of esophageal function (FLIP, HRM, and a combined FLIP and HRM with neuromyogenic [NM] classification (44)), we performed ordinal logistic regression that modeled likelihoods of increasing clinical severity. For this analysis, we excluded SSc patients with achalasia or esophagogastric junction outflow obstruction. For FLIP we combined the “borderline/diminished” and “impaired/disordered” phenotypes. For NM we combined the Stage I and Stage II ineffective motility phenotypes. The resultant ordering for the ordinal regression was “normal” < “ineffective” < “absent” for HRM; “normal” < “borderline/diminished & impaired/disordered” < “absent” for FLIP; and “normal” < “stages I & II – ineffective” < “stage III – absent” for NM.

For association testing with quantitative clinical measures in SSc, we first performed a principal components analysis (PCA) on a set of nine quantitative clinical traits: HRM mean distal contractile integral (DCI), HRM basal esophagogastric junction (EGJ) pressure, HRM EGJ contractile index, HRM median integrated relaxation pressure (IRP), FLIP intra-balloon pressure at 60ml, FLIP EGJ distensibility index, the GerdQ impact score, the BEDQ score, and the NEQOL score. Missing values (6/90, 6.7%) were imputed using the mean across all patients; sensitivity analysis using median imputation produced nearly identical PCs (PC1: r=0.9996; PC2: r=0.9982). We then modeled associations with the first two PCs using linear regression. Gene expression was represented using log-transformed counts.

SUPPLEMENTAL FIGURES

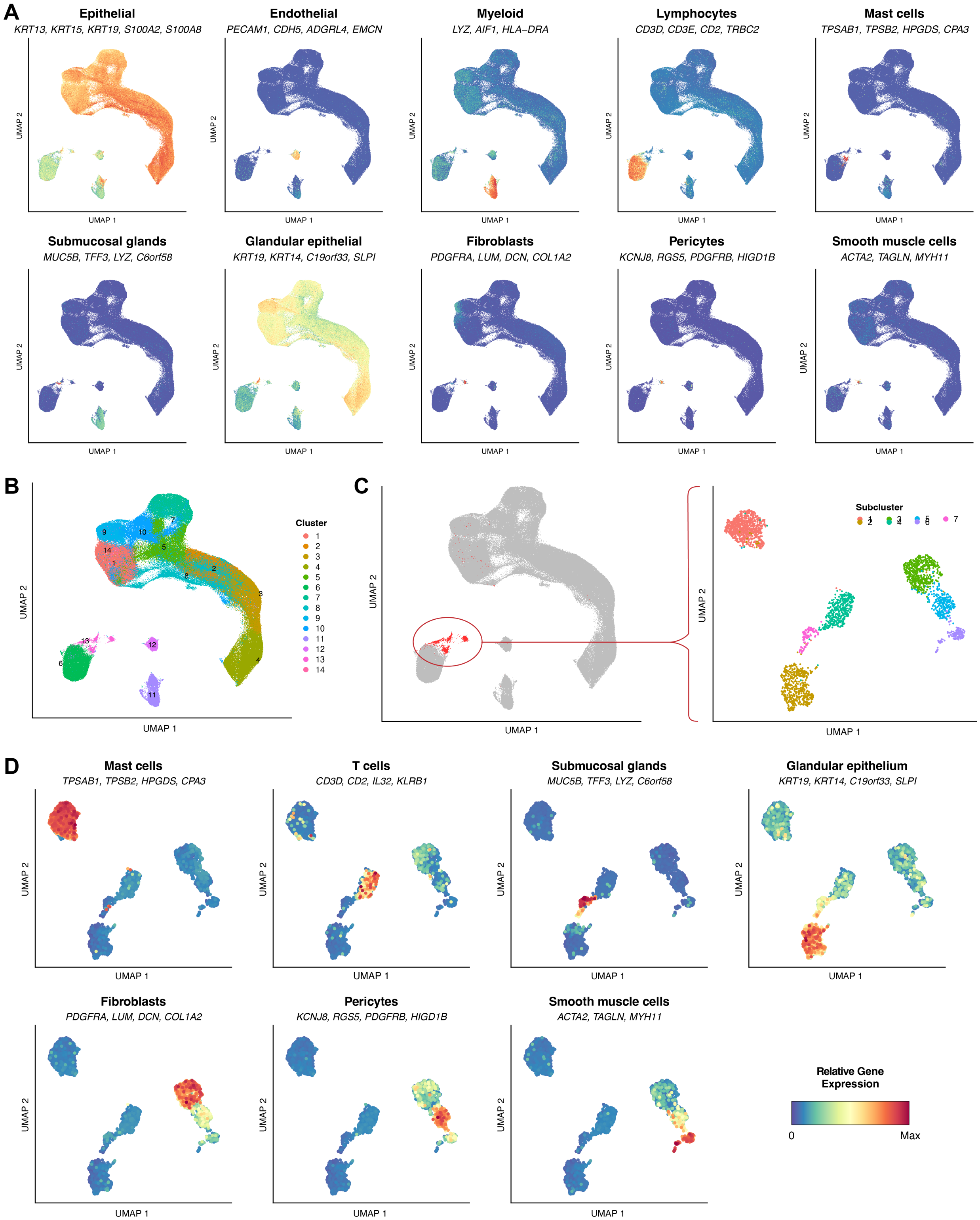

Supplemental Figure 1. Cell clustering and cell type annotation. A) Cell type module scores for 10 detected cell types, colored by relative gene expression of the listed genes. **B)** Projected UMAP embeddings calculated on the top 40 PCs with n=200 neighbors. Cells are colored by cluster, which are numbered in descending order by total cell count. Clusters were determined using Seurat's modularity-based clustering on a shared nearest neighbor (SNN) graph with k=25 and resolution=0.25 on the top 40 PCs. **C)** Isolation of cluster 13 for annotation of cell types with smaller proportions of cells and projected UMAP embeddings for cluster 13, colored by subcluster. **D)** Cell type module scores for 7 detected cell types in subcluster 13, colored by relative gene expression of the listed genes.

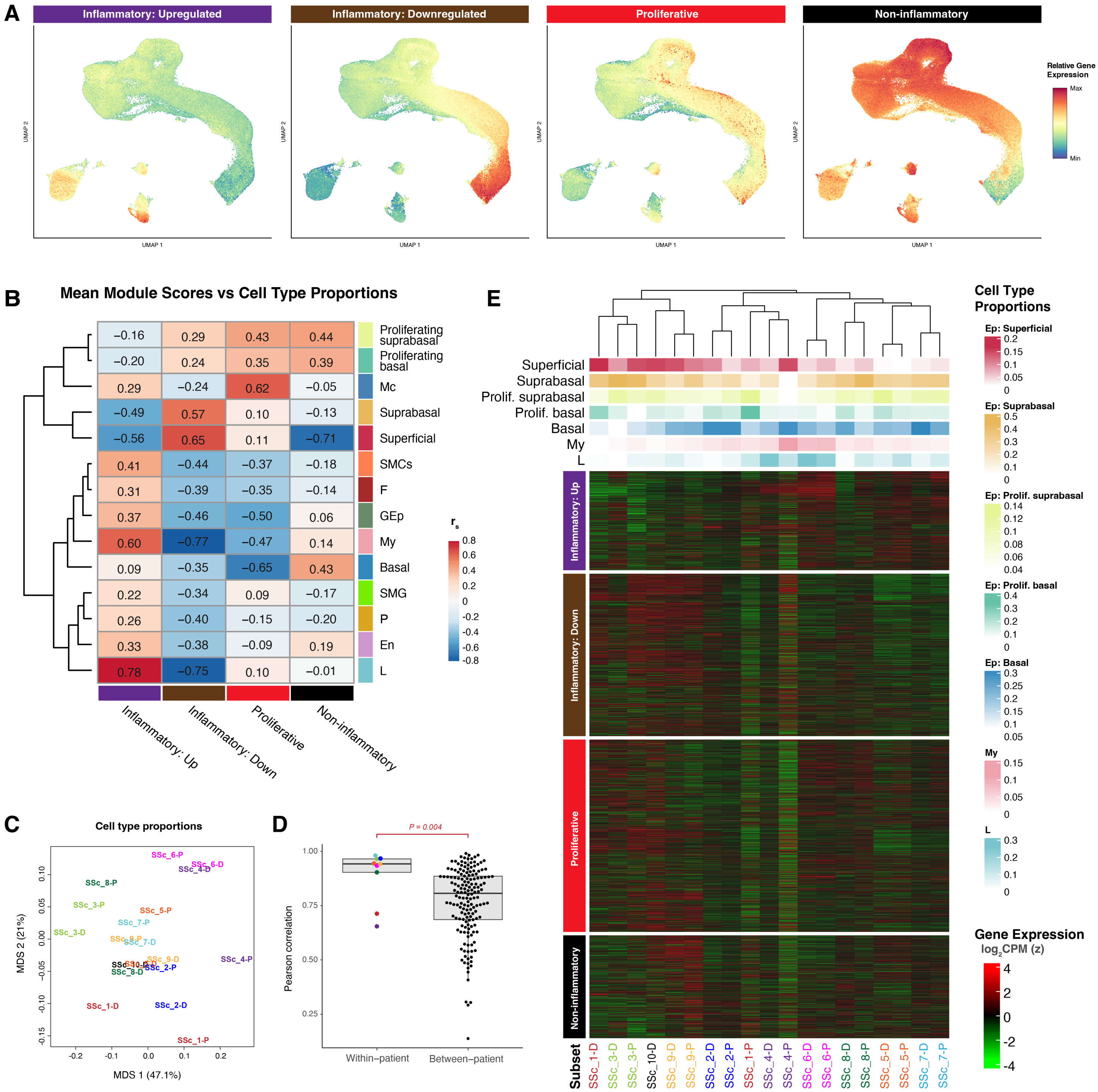

Supplemental Figure 2. Single-cell expression of intrinsic molecular subsets in SSc. A) EEC UMAPs colored by relative expression of “intrinsic” gene expression signatures for previously reported esophageal patient subsets identified via bulk RNA-seq (from Taroni *et al*. 2015, Additional file 11). **B)** Spearman correlations between mean intrinsic gene expression signature module scores and cell type proportions, aggregated by sample, and hierarchically clustered (Ward) by cell-type-specific correlation patterns. **C)** Multidimensional scaling (MDS) plot of sample aggregate cell type proportions. **D)** Cell type proportion correlations between biopsy locations within the same patients (left) and between all other sample combinations (right). **E)** Heatmap of log scaled pseudobulk gene expression counts for differentially expressed genes between intrinsic subsets (from Taroni *et al*. 2015, Additional file 11). Samples are hierarchically clustered by average linkage of Pearson correlation distances. Cell type proportions for epithelial and myeloid and lymphoid cells are visualized for each sample. En, endothelial; Ep, epithelial; F, fibroblast; L, lymphocytes; Mc, mast cells; My, myeloid; P, pericytes; SMCs, smooth muscle cells; SMG, submucosal glands.

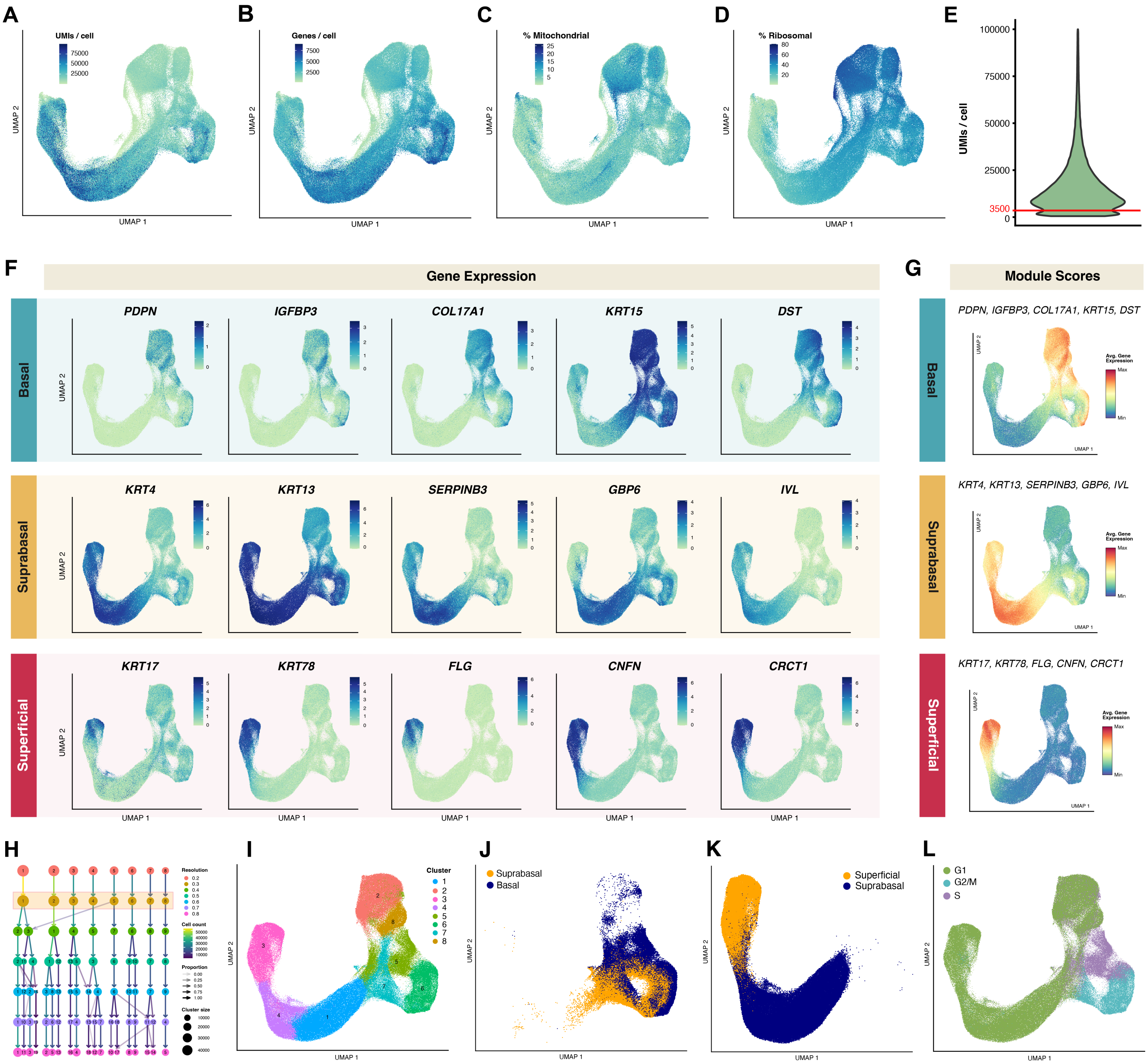

Supplemental Figure 3. Epithelial cell quality control and annotation. A) EEC UMAP projection, colored by the number of unique molecular identifiers (UMIs) per cell. **B)** EEC UMAP projection, colored by the number of detected genes per cell. **C)** EEC UMAP projection, colored by the number of proportion of UMIs mapping to mitochondrial DNA. **D)** EEC UMAP projection, colored by the proportion of UMIs mapping to ribosomal DNA. **E)** Distribution of number of UMIs detected per cell. EECs with <3500 UMIs/cell were removed from further analysis. **F)** EEC UMAPs colored by gene expression (log(counts+1))) for genes included in compartment-specific expression scores. **G)** EEC UMAPs colored by compartment expression scores. **H)** Clustree graph of clustering results at different resolutions. A resolution of 0.3 was chosen. **I)** Projected UMAP embeddings for post-quality-control EECs calculated on the top 35 PCs with n=100 neighbors. Cells are colored by cluster, which are numbered in descending order by total cell count. Clusters were determined using Seurat's modularity-based clustering on a SNN graph with k=50 and resolution=0.25 on the top 35 PCs. **J)** Result of K-means clustering to distinguish suprabasal from basal cells, based on expression of corresponding cluster markers (**F**) in clusters 3, 4, and 5, with K=2. **K)** Result of K-means clustering to distinguish superficial from suprabasal cells, based on expression of corresponding cluster markers (**F**) in clusters 6, 7, and 8, with K=2. **L)** Predicted classification of cell cycle state in EECs determined using Seurat's CellCycleScoring() function.

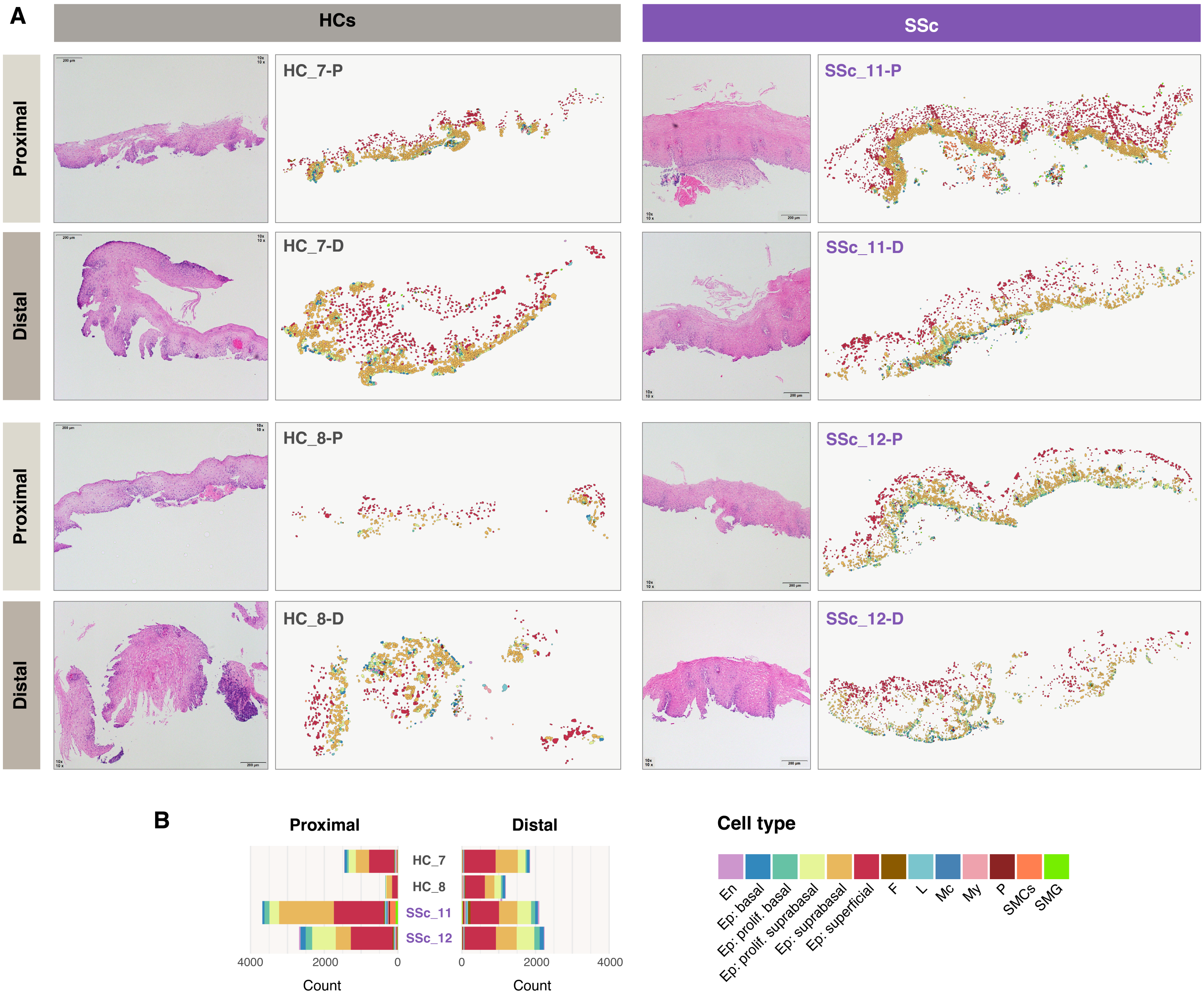

Supplemental Figure 4. Spatial molecular imaging of esophageal mucosa. A) Adjacent esophageal mucosa tissue slides either stained with hematoxylin and eosin (H&E) or analyzed by CosMx spatial molecular imaging are shown with annotations conferred from scRNA-seq label transfer for two healthy controls (HCs) and two SSc patients with absent contractility. **B)** Spatial molecular imaging cell type counts are shown by individual and biopsy location. En, endothelial; Ep, epithelial; F, fibroblast; L, lymphocytes; Mc, mast cells; My, myeloid; P, pericytes; SMCs, smooth muscle cells; SMG, submucosal glands.

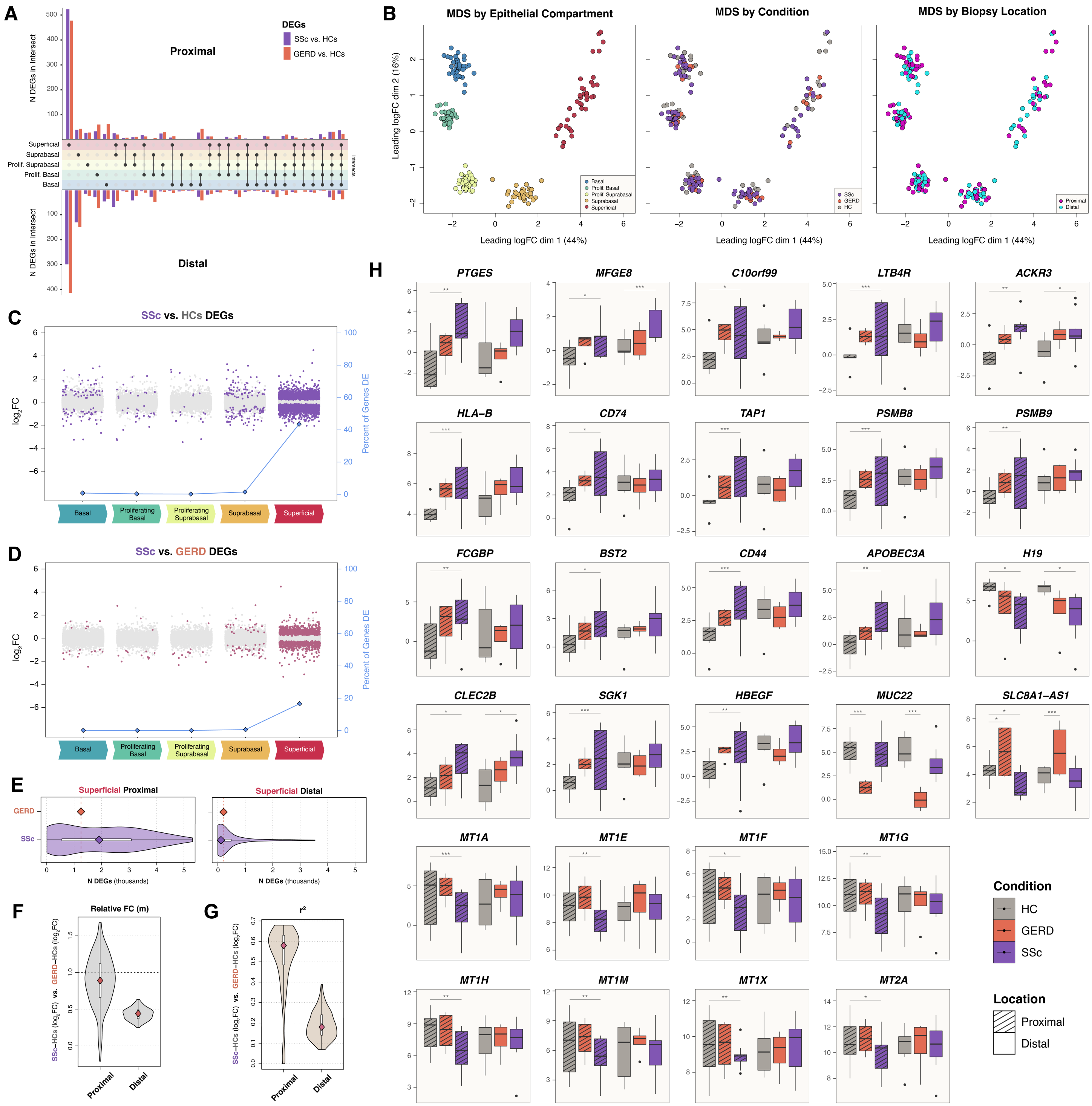

Supplemental Figure 5. Differential gene expression patterns between conditions in esophageal epithelial cells. A) UpSet plots showing differential gene expression at the single-cell level by epithelial compartment for SSc vs HCs (purple) and GERD vs HCs (orange) in the proximal and distal esophagus. **B)** Multidimensional scaling plots of aggregate gene expression samples colored by sample, epithelial compartment, condition, and biopsy location. **C-D)** Pseudobulk gene expression differences between SSc and HCs **(C)** and between SSc and GERD **(D)** are shown for each EEC compartment. Significant DEGs (FDR<0.05) are highlighted in purple and raspberry, respectively. The percentages of genes that were significantly differentially expressed in each compartment are plotted in blue in along the secondary Y axis. **E)** The number of significant DEGs are shown for GERD vs. HCs (orange) and for all permutations of n=4 samples for SSc vs. HCs (purple) in superficial cells for both proximal and distal regions. The point within the SSc distribution is the median value. **F-G)** The distributions of relative FC or slope m **(F)** and coefficient of determination r^2 **(G)** are shown for all permutations of n=4 SSc samples, determined by modeling a linear regression with intercept=0 for the $\log_2$FC of SSc vs. HCs against the $\log_2$FC of GERD vs. HCs for all expressed genes in the superficial compartment for both proximal and distal regions. DEG, differentially expressed gene; FC, fold change. **H)** Boxplots of pseudobulk gene expression, aggregated by condition, biopsy location, and EEC compartment for disease-specific DEGs referenced in the main text. Pairwise differential expression was determined using edgeR's quasi-likelihood F-tests: *P<0.05; **P<0.05; ***P<0.0005.

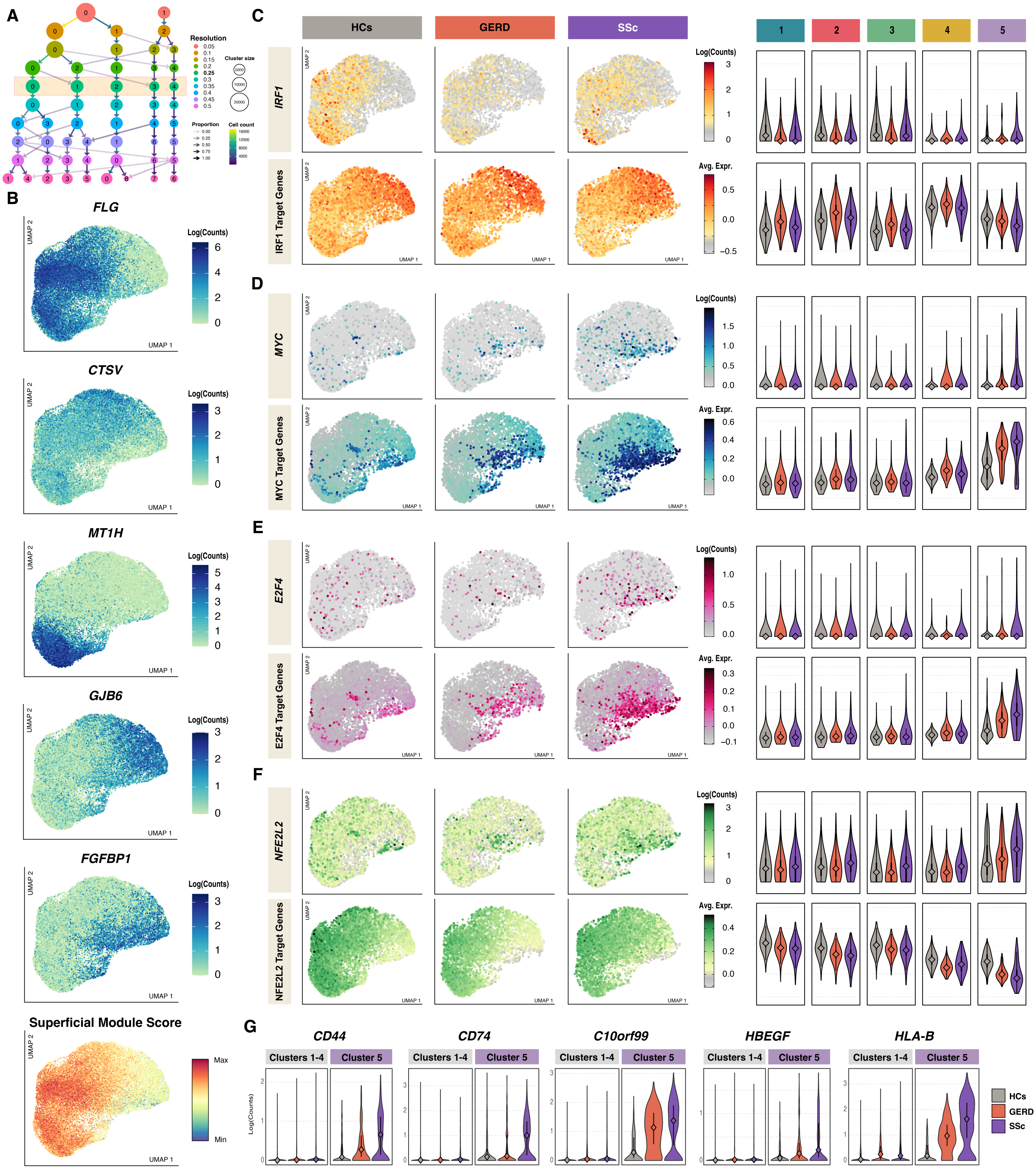

Supplemental Figure 6. Expression of cluster markers, enriched transcription factors, and their targets in superficial EECs in proximal esophagus. A) Clustree graph of clustering results at different resolutions. A resolution of 0.25 was chosen. **B)** UMAPs with expression for the top superficial cluster markers are shown along with the epithelial differentiation score for superficial cells. **C-E)** The distributions of enriched transcription factor expression and target module scores are shown proximal, superficial cells by condition and superficial cluster for **C)** *IRF1*, **D)** *MYC*, **E)** *E2F4*, and **F)** *NE2L2*. **G)** Violin plots of gene expression in superficial cluster 5 vs. all other superficial subclusters for SSc-specific differentially expressed genes that were uniquely expressed in cluster 5 within superficial EECs.

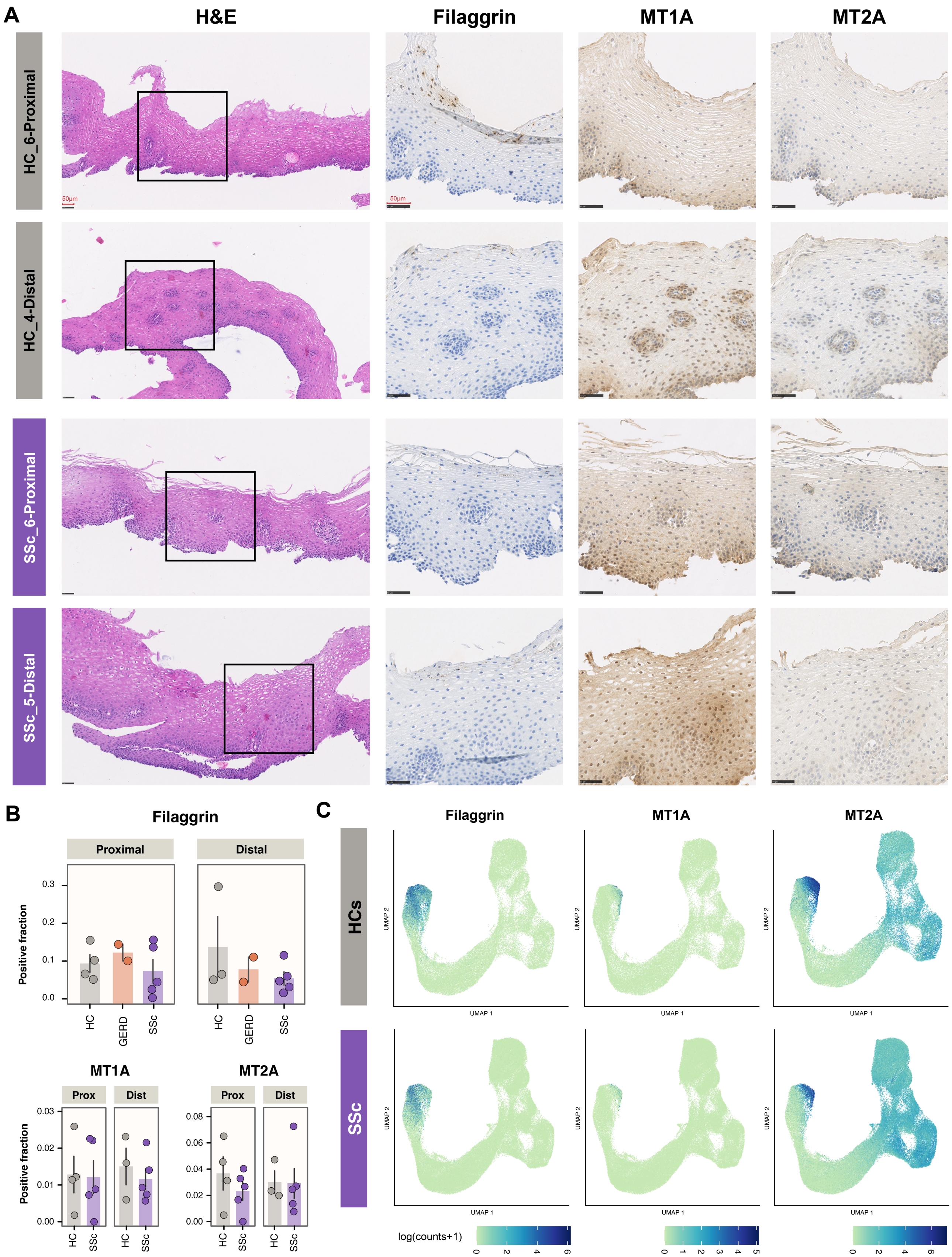

Supplemental Figure 7. Immunohistochemistry. A) Representative images of H&E and immunohistochemistry (IHC; filaggrin, MT1A, and MT2A) tissue sections from proximal and distal esophageal mucosal biopsies from a healthy control and an SSc patient. **B)** Proportions of 25 µm tiles expressing filaggrin, MT1A, and MT2A within each IHC image, grouped by condition and biopsy location. Bar heights denote the mean with the vertical lines indicating the standard error. **C)** EEC UMAPs colored by gene expression (log(counts+1)) for *FLG*, *MT1A*, and *MT2A* genes, grouped by condition, for HCs and SSc patient samples.

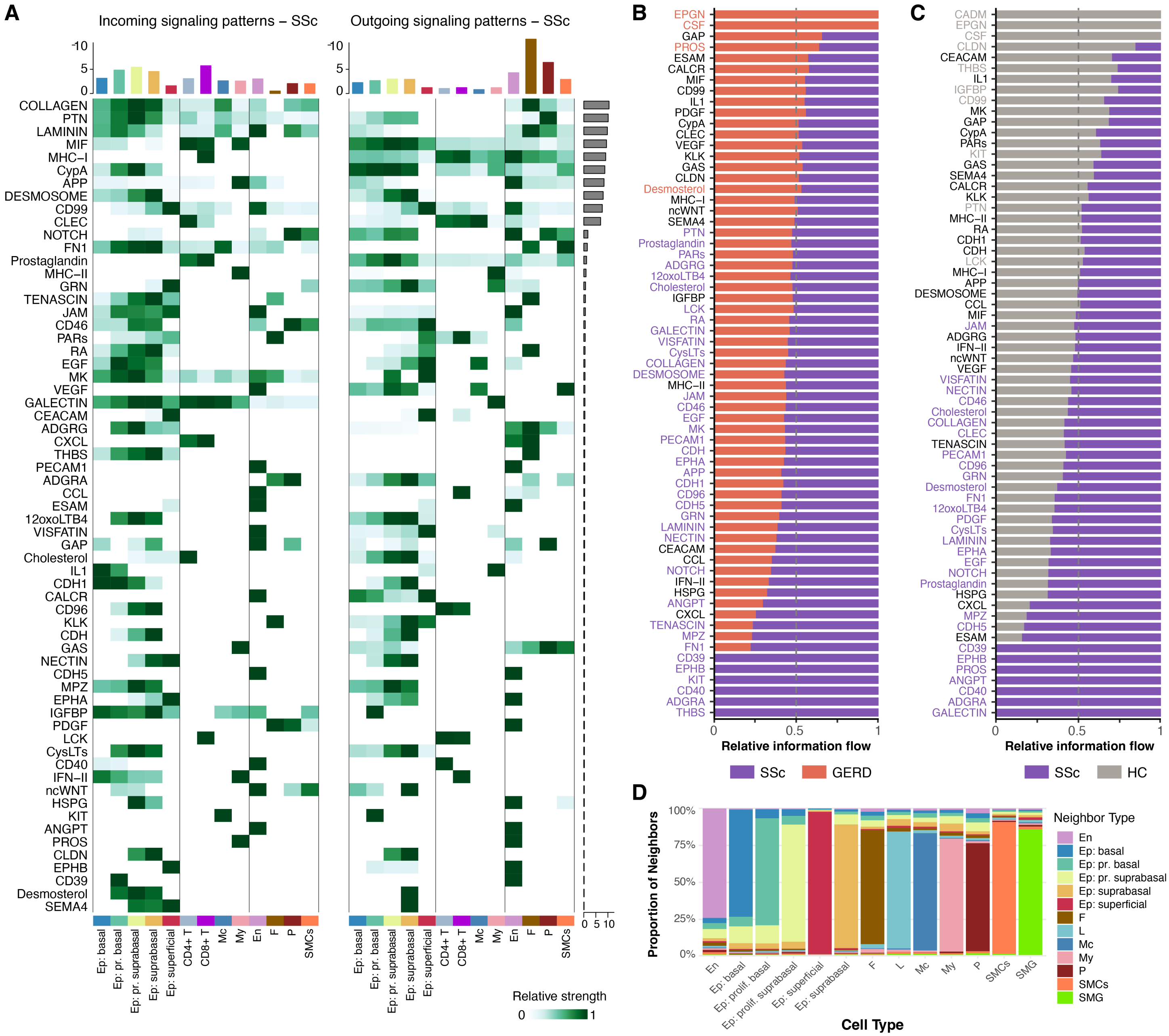

Supplemental Figure 8. Pathway-level signaling patterns in SSc. A) Relative contribution of cell types to incoming (left) and outgoing (right) signaling across all detected pathways in SSc. Heatmap values are row-scaled to show relative signaling strength across cell types for each pathway. Top bars indicate aggregate signaling strength per cell type; right bars indicate aggregate signaling strength per pathway. **B)** Comparison of relative information flow for signaling pathways between SSc and GERD. Pathway labels are colored by the condition with significantly greater information flow based on paired Wilcoxon test ($P < 0.05$); black labels indicate no significant difference. **C)** Comparison of relative information flow for signaling pathways between SSc and HCs. **D)** Spatial neighborhood composition showing the proportion of each cell type among neighbors, by cell type, in SSc.

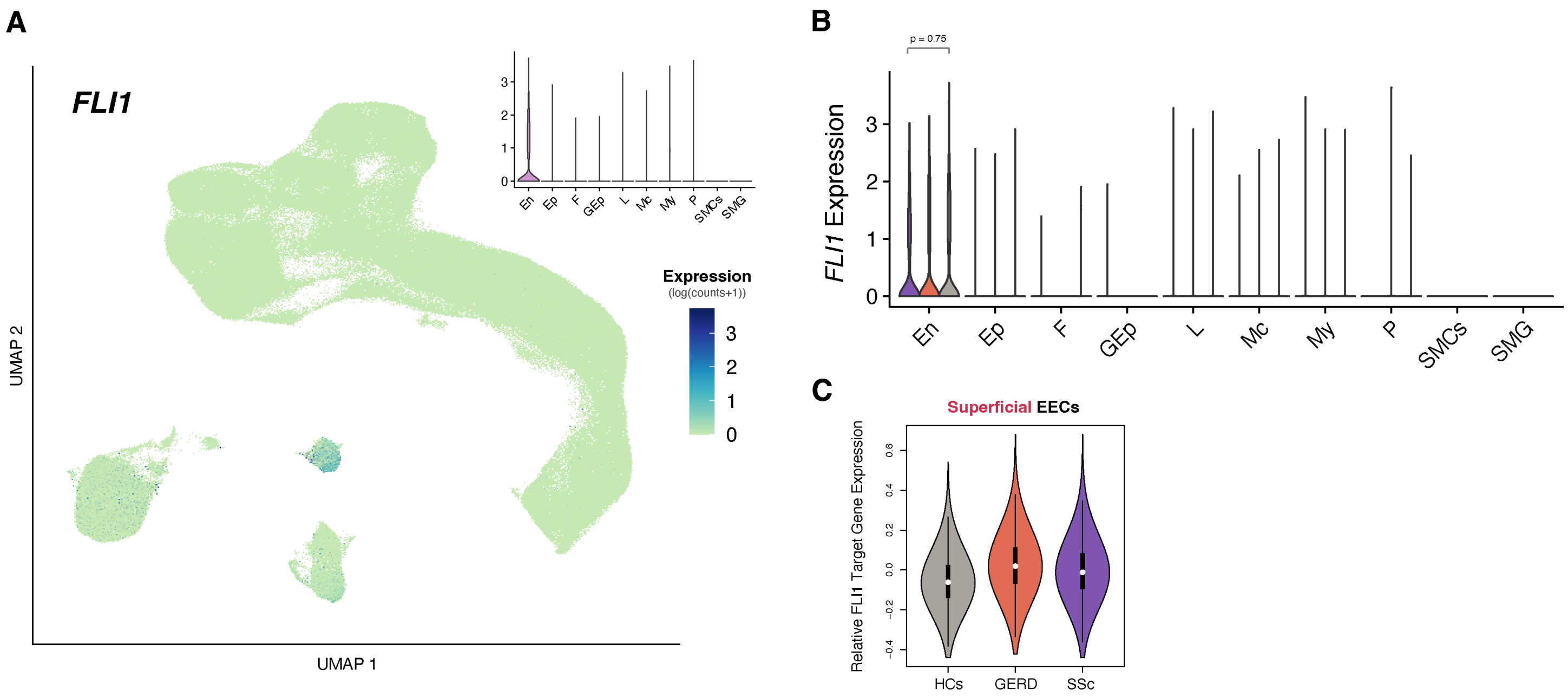

Supplemental Figure 9. *FLI1* expression in human esophageal mucosa. A) UMAP of integrated mucosal dataset, colored by *FLI1* expression. The distribution of *FLI1* expression by cell type is shown in the upper right. **B)** Distribution of *FLI1* expression by cell type and disease state. Endothelial cells had the highest *FLI1* expression, but the expression differences between diseases was not significant. **C)** Violin plot of the relative expression of FLI1 target genes in superficial cells.

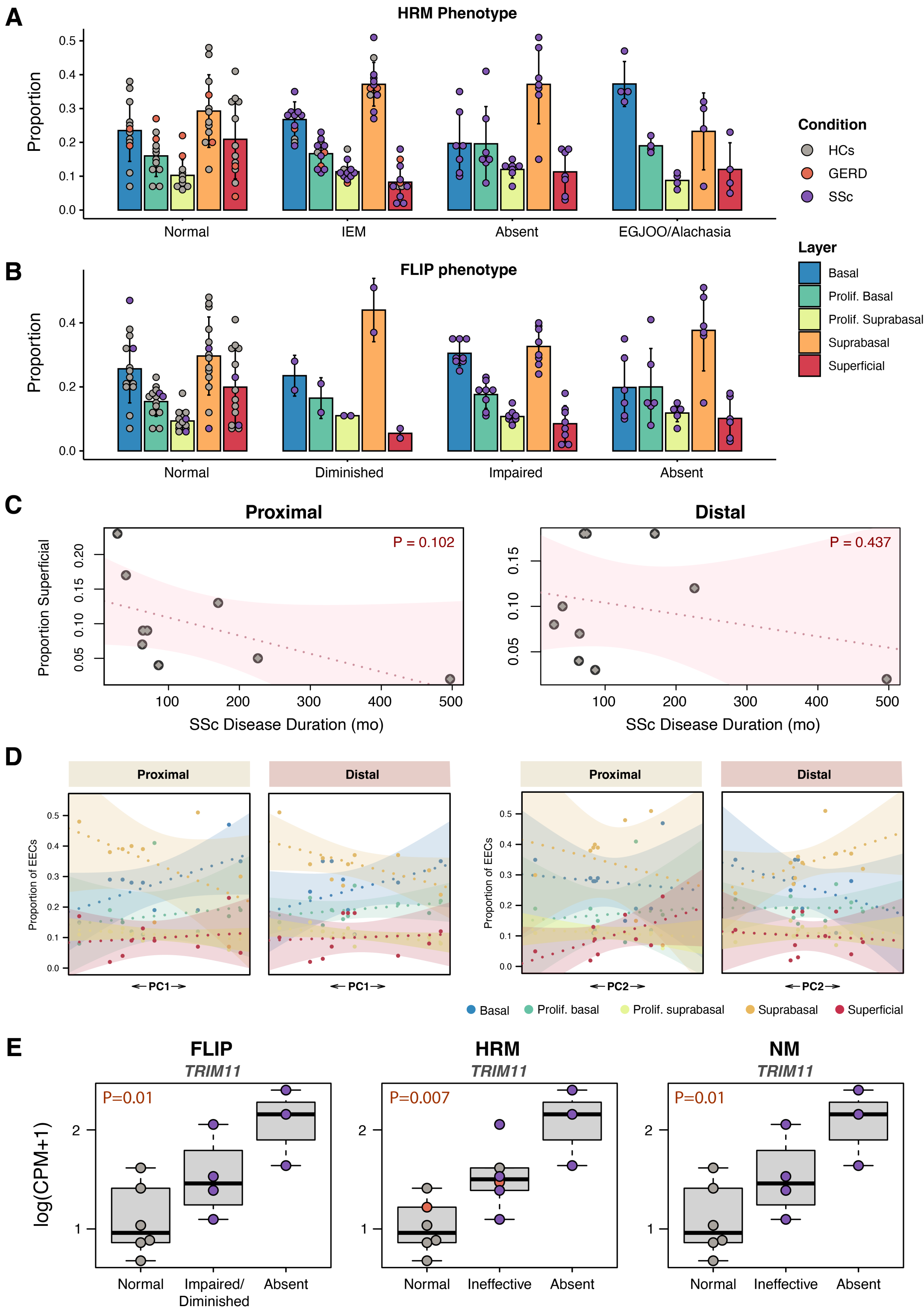

Supplemental Figure 10. Epithelial cell compartment proportions and additional correlations by motility phenotype. A-B) Proportions of esophageal epithelial cell compartments by HRM **(A)** and FLIP **(B)** esophageal dysmotility phenotypes are shown for the proximal and distal esophagus samples. The bars denote the mean values, the vertical lines the standard deviations, and the points the individual sample proportions. **C)** The relationship between the proportion of superficial cells and the SSc disease duration is plotted for proximal and distal samples. Trendlines with 95% confidence intervals are plotted in pink, and correlation P-values are displayed in the upper right corners. **D)** Correlations between EEC compartments and clinical trait PCs in SSc cases. Linear regression trendlines with 95% confidence intervals are shown for each compartment against PC1 and PC2. **E)** The distribution of normalized gene expression values by esophageal motility phenotype are shown for the *TRIM11* gene for the FLIP, HRM, and NM esophageal dysmotility classifications. Unadjusted ordinal regression P-values are displayed in the upper left. *TRIM11* was the most strongly association gene with the dysmotility phenotypes by P-value. HRM, high-resolution manometry; FLIP, functional luminal imaging probe panometry; IEM, ineffective esophageal motility; EGJOO, esophagogastric junction outflow obstruction; NM, neuromyogenic model.

SUPPLEMENTAL REFERENCES

1. Carlson DA, et al. Heterogeneity of primary and secondary peristalsis in systemic sclerosis: A new model of "scleroderma esophagus". *Neurogastroenterol Motil.* 2022;34(7):e14284.
2. Clevenger MH, et al. Suprabasal cells retain progenitor cell identity programs in eosinophilic esophagitis-driven basal cell hyperplasia. *JCI Insight.* 2023;8(19).
3. Carlson DA, et al. Classifying Esophageal Motility by FLIP Panometry: A Study of 722 Subjects With Manometry. *Am J Gastroenterol.* 2021;116(12):2357-66.
4. Koop AH, et al. The impact of primary peristalsis, contractile reserve, and secondary peristalsis on esophageal clearance measured by timed barium esophagogram. *Neurogastroenterol Motil.* 2023:e14638.
5. Stern EK, et al. Abnormal esophageal acid exposure on high-dose proton pump inhibitor therapy is common in systemic sclerosis patients. *Neurogastroenterol Motil.* 2018;30(2).
6. Yadlapati R, et al. Esophageal motility disorders on high-resolution manometry: Chicago classification version 4.0((c)). *Neurogastroenterol Motil.* 2021;33(1):e14058.
7. Rooney KP, et al. Esophagogastric Junction Opening Parameters Are Consistently Abnormal in Untreated Achalasia. *Clin Gastroenterol Hepatol.* 2021;19(5):1058-60.e1.
8. Carlson DA, et al. Evaluating esophageal motility beyond primary peristalsis: Assessing esophagogastric junction opening mechanics and secondary peristalsis in patients with normal manometry. *Neurogastroenterol Motil.* 2021;33(10):e14116.
9. Carlson DA, et al. Normal Values of Esophageal Distensibility and Distension-Induced Contractility Measured by Functional Luminal Imaging Probe Panometry. *Clin Gastroenterol Hepatol.* 2019;17(4):674-81 e1.
10. Jonasson C, et al. Validation of the GerdQ questionnaire for the diagnosis of gastro-oesophageal reflux disease. *Aliment Pharmacol Ther.* 2013;37(5):564-72.
11. Taft TH, et al. Development and validation of the brief esophageal dysphagia questionnaire. *Neurogastroenterol Motil.* 2016;28(12):1854-60.
12. Bedell A, et al. Development of the Northwestern Esophageal Quality of Life Scale: A Hybrid Measure for Use Across Esophageal Conditions. *Am J Gastroenterol.* 2016;111(4):493-9.
13. Hao Y, et al. Integrated analysis of multimodal single-cell data. *Cell.* 2021;184(13):3573-87 e29.
14. Hippen AA, et al. miQC: An adaptive probabilistic framework for quality control of single-cell RNA-sequencing data. *PLoS Comput Biol.* 2021;17(8):e1009290.
15. Germain PL, et al. Doublet identification in single-cell sequencing data using scDblFinder. *F1000Res.* 2021;10:979.
16. Hafemeister C, and Satija R. Normalization and variance stabilization of single-cell RNA-seq data using regularized negative binomial regression. *Genome Biol.* 2019;20(1):296.
17. Lause J, et al. Analytic Pearson residuals for normalization of single-cell RNA-seq UMI data. *Genome Biol.* 2021;22(1):258.
18. McInnes L, et al.; 2018:arXiv:1802.03426.
19. Taroni JN, et al. Molecular characterization of systemic sclerosis esophageal pathology identifies inflammatory and proliferative signatures. *Arthritis Res Ther.* 2015;17:194.
20. Oh S, et al. HGNChelper: identification and correction of invalid gene symbols for human and mouse [version 1; peer review: 2 approved, 1 approved with reservations]. *F1000Research.* 2020;9(1493).
21. Robinson MD, et al. edgeR: a Bioconductor package for differential expression analysis of digital gene expression data. *Bioinformatics.* 2010;26(1):139-40.
22. Busslinger GA, et al. Human gastrointestinal epithelia of the esophagus, stomach, and duodenum resolved at single-cell resolution. *Cell Rep.* 2021;34(10):108819.

23. Kabir MF, et al. Single cell transcriptomic analysis reveals cellular diversity of murine esophageal epithelium. *Nat Commun.* 2022;13(1):2167.
24. Okumura T, et al. Neurotrophin receptor p75(NTR) characterizes human esophageal keratinocyte stem cells in vitro. *Oncogene.* 2003;22(26):4017-26.
25. Rochman M, et al. Single-cell RNA-Seq of human esophageal epithelium in homeostasis and allergic inflammation. *JCI Insight.* 2022;7(11).
26. Tirosh I, et al. Dissecting the multicellular ecosystem of metastatic melanoma by single-cell RNA-seq. *Science.* 2016;352(6282):189-96.
27. Fonseka CY, et al. Mixed-effects association of single cells identifies an expanded effector CD4(+) T cell subset in rheumatoid arthritis. *Sci Transl Med.* 2018;10(463).
28. Mootha VK, et al. PGC-1alpha-responsive genes involved in oxidative phosphorylation are coordinately downregulated in human diabetes. *Nat Genet.* 2003;34(3):267-73.
29. Subramanian A, et al. Gene set enrichment analysis: a knowledge-based approach for interpreting genome-wide expression profiles. *Proc Natl Acad Sci U S A.* 2005;102(43):15545-50.
30. Liberzon A, et al. The Molecular Signatures Database (MSigDB) hallmark gene set collection. *Cell Syst.* 2015;1(6):417-25.
31. Ritchie ME, et al. limma powers differential expression analyses for RNA-sequencing and microarray studies. *Nucleic Acids Res.* 2015;43(7):e47.
32. Chen Y, et al. From reads to genes to pathways: differential expression analysis of RNA-Seq experiments using Rsubread and the edgeR quasi-likelihood pipeline. *F1000Res.* 2016;5:1438.
33. McCarthy DJ, et al. Differential expression analysis of multifactor RNA-Seq experiments with respect to biological variation. *Nucleic Acids Res.* 2012;40(10):4288-97.
34. Wu T, et al. clusterProfiler 4.0: A universal enrichment tool for interpreting omics data. *Innovation (Camb).* 2021;2(3):100141.
35. Consortium EP. An integrated encyclopedia of DNA elements in the human genome. *Nature.* 2012;489(7414):57-74.
36. Lachmann A, et al. ChEA: transcription factor regulation inferred from integrating genome-wide ChIP-X experiments. *Bioinformatics.* 2010;26(19):2438-44.
37. Kou Y, et al. Berlin, Heidelberg: Springer Berlin Heidelberg; 2013:416-30.
38. Chen EY, et al. Enrichr: interactive and collaborative HTML5 gene list enrichment analysis tool. *BMC Bioinformatics.* 2013;14:128.
39. Bankhead P, et al. QuPath: Open source software for digital pathology image analysis. *Sci Rep.* 2017;7(1):16878.
40. Achanta R, et al. SLIC superpixels compared to state-of-the-art superpixel methods. *IEEE Trans Pattern Anal Mach Intell.* 2012;34(11):2274-82.
41. Aran D, et al. Reference-based analysis of lung single-cell sequencing reveals a transitional profibrotic macrophage. *Nat Immunol.* 2019;20(2):163-72.
42. Jefferis G, et al. RANN: Fast Nearest Neighbour Search (Wraps ANN Library) Using L2 Metric. 2024.
43. Jin S, et al. CellChat for systematic analysis of cell-cell communication from single-cell transcriptomics. *Nat Protoc.* 2025;20(1):180-219.
44. Koop AH, et al. The impact of primary peristalsis, contractile reserve, and secondary peristalsis on esophageal clearance measured by timed barium esophagogram. *Neurogastroenterol Motil.* 2023;35(10):e14638.
